# Differential chromatin accessibility and transcriptional dynamics define breast cancer subtypes and their lineages

**DOI:** 10.1038/s43018-024-00773-6

**Published:** 2024-10-30

**Authors:** Michael D. Iglesia, Reyka G. Jayasinghe, Siqi Chen, Nadezhda V. Terekhanova, John M. Herndon, Erik Storrs, Alla Karpova, Daniel Cui Zhou, Nataly Naser Al Deen, Andrew T. Shinkle, Rita Jui-Hsien Lu, Wagma Caravan, Andrew Houston, Yanyan Zhao, Kazuhito Sato, Preet Lal, Cherease Street, Fernanda Martins Rodrigues, Austin N. Southard-Smith, André Luiz N. Targino da Costa, Houxiang Zhu, Chia-Kuei Mo, Lisa Crowson, Robert S. Fulton, Matthew A. Wyczalkowski, Catrina C. Fronick, Lucinda A. Fulton, Hua Sun, Sherri R. Davies, Elizabeth L. Appelbaum, Sara E. Chasnoff, Madelyn Carmody, Candace Brooks, Ruiyang Liu, Michael C. Wendl, Clara Oh, Diane Bender, Carlos Cruchaga, Oscar Harari, Andrea Bredemeyer, Kory Lavine, Ron Bose, Julie Margenthaler, Jason M. Held, Samuel Achilefu, Foluso Ademuyiwa, Rebecca Aft, Cynthia Ma, Graham A. Colditz, Tao Ju, Stephen T. Oh, James Fitzpatrick, E. Shelley Hwang, Kooresh I. Shoghi, Milan G. Chheda, Deborah J. Veis, Feng Chen, Ryan C. Fields, William E. Gillanders, Li Ding

**Affiliations:** 1 Department of Medicine, Washington University in St. Louis, St. Louis, MO USA; 2 McDonnell Genome Institute, Washington University in St. Louis, St. Louis, MO USA; 3 Department of Surgery, Washington University in St. Louis, St. Louis, MO USA; 4 Siteman Cancer Center, Washington University in St. Louis, St. Louis, MO USA; 5 Department of Mathematics, Washington University in St. Louis, St. Louis, MO USA; 6 Department of Genetics, Washington University in St. Louis, St. Louis, MO USA; 7 Bursky Center for Human Immunology & Immunotherapy, Washington University in St. Louis, St. Louis, MO USA; 8 Department of Psychiatry, Washington University in St. Louis, St. Louis, MO USA; 9 Department of Pathology and Immunology, Washington University in St. Louis, St. Louis, MO USA; 10 Department of Developmental Biology, Washington University in St. Louis, St. Louis, MO USA; 11 Department of Radiology, Washington University in St. Louis, St. Louis, MO USA; 12 John Cochran Veterans Hospital, St. Louis, MO USA; 13 Division of Public Health Sciences, Washington University School of Medicine, St. Louis, MO USA; 14 Department of Computer Science and Engineering, Washington University in St. Louis, St. Louis, MO USA; 15 Washington University Center for Cellular Imaging, Washington University in St. Louis, St. Louis, MO USA; 16 Departments of Neuroscience and Cell Biology & Physiology, Washington University in St. Louis, St. Louis, MO USA; 17 Department of Surgery, Duke University Medical Center, Durham, NC England

**Keywords:** Breast cancer, Cancer genomics

## Abstract

Breast cancer (BC) is defined by distinct molecular subtypes with different cells of origin. The transcriptional networks that characterize the subtype-specific tumor-normal lineages are not established. In this work, we applied bulk, single-cell and single-nucleus multi-omic techniques as well as spatial transcriptomics and multiplex imaging on 61 samples from 37 patients with BC to show characteristic links in gene expression and chromatin accessibility between BC subtypes and their putative cells of origin. Regulatory network analysis of transcription factors underscored the importance of BHLHE40 in luminal BC and luminal mature cells and KLF5 in basal-like tumors and luminal progenitor cells. Furthermore, we identify key genes defining the basal-like (*SOX6* and *KCNQ3*) and luminal A/B (*FAM155A* and *LRP1B*) lineages. Exhausted CTLA4-expressing CD8^+^ T cells were enriched in basal-like BC, suggesting an altered means of immune dysfunction. These findings demonstrate analysis of paired transcription and chromatin accessibility at the single-cell level is a powerful tool for investigating cancer lineage and highlight transcriptional networks that define basal and luminal BC lineages.

## Abstract (web summary)

Ding and colleagues use bulk, single-cell and single-nucleus multi-omics together with spatial transcriptomics and multiplex imaging of clinical tumor samples to characterize gene expression and chromatin accessibility of breast cancer lineages.

## Main

**Fig. 1:**
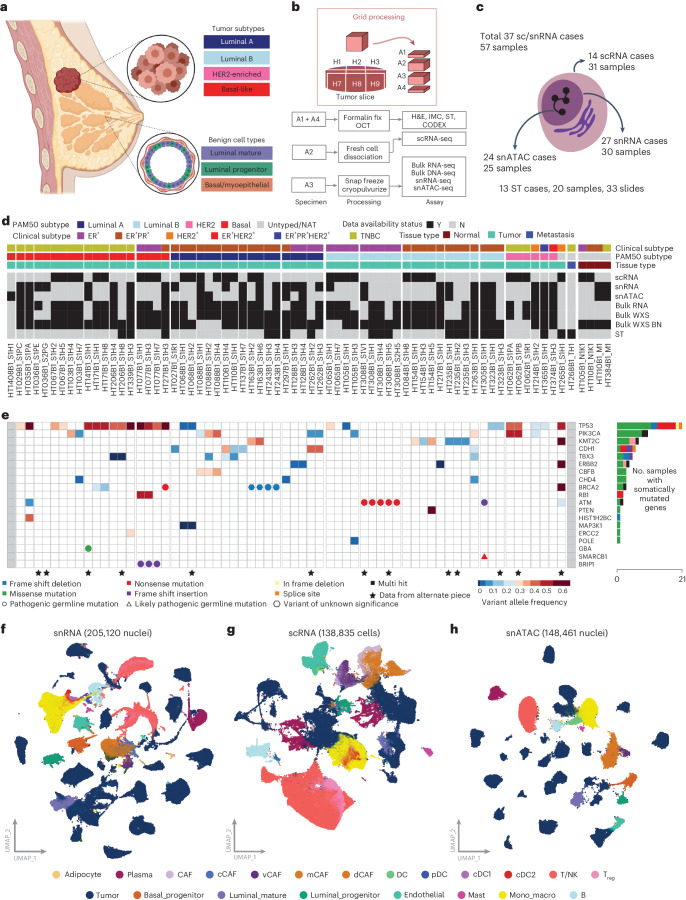
Study design, data collected and genomic alterations. **a**, Summary of benign breast duct cell types and BC subtypes. The image was created with BioRender.com. **b**, Sample grid processing method utilized in the study to perform various assays on each tumor sample systematically. **c**, Summary of data types available for single-cell, single-nucleus and ST processing. **d**, Data overview of the cohort of 61 samples. The N1K1 and M1 suffix denotes normal adjacent tumor samples. Clinical characteristics and data type availability are shown for each tumor piece. Data types include scRNA-seq, snRNA-seq, snATAC-seq, bulk-RNA-seq, ST and bulk WES of tumor and blood normal (BN). **e**, Genomic landscape of the sample cohort showing the top significantly mutated genes. Color scale in heatmap denotes VAF for each gene. All mutations are somatic, unless indicated by a colored circle/triangle/pentagon designating germline variants of different annotated significance. **f**, Uniform Manifold Approximation and Projection (UMAP) plots of all cell types for snRNA-seq data colored by cell types. **g**, UMAP plots of all cell types for single-cell RNA data colored by cell types. **h**, UMAP plots of all cell types for snATAC-seq data colored by cell types.

BC is the most common cancer in women, with 2.1 million new cases diagnosed in 2018 (ref. ^1^). Treatment is guided by biomarker profiles, specifically the expression of estrogen receptor (ER), progesterone receptor (PR) and human epidermal growth factor receptor 2 (HER2), which approximate the BC molecular subtypes^2^. Breast ductal epithelium, from which BC arises, is divided into two main lineages (Fig. 1a). Luminal cells line the interior of the breast duct and are surrounded by a layer of thin, contractile basal myoepithelial cells. Both luminal and basal cells are derived from a long-lived, bipotent mammary stem cell and more differentiated unipotent progenitor cells exist within the basal and luminal lineages to renew these compartments in the breast duct^3,4^. Several groups have interrogated normal and BC cell types at the single-cell level, further refining our understanding of the expression profiles associated with these cell lineages^5–18^.

In the healthy breast duct, distinct transcriptional programs assign cells to a luminal or basal fate. The regulatory network of *GATA3*, *FOXA1* and the ER *ESR1* represent a signaling axis that is essential for the maturation of luminal breast cells and the development of luminal BC^19–22^. The ETS-domain transcription factor (TF) ELF5 is a key determinant of luminal cell fate and the secretory sublineage of luminal cells^23,24^. Basal breast cells, on the other hand, maintain a more mesenchymal state, with p63 and SOX family TFs playing key roles in the maintenance of basal cell fate^25,26^. Transcriptional programs and chromatin accessibility patterns are useful as markers of cell lineage and cell of origin^27–30^. The structure and chromatin accessibility of mouse breast tissue has established distinct features of cell states and underscored the utility of chromatin accessibility as a marker of breast cell lineage^31,32^. Chromatin accessibility has also identified master regulators such as SOX10, which regulate the transition from benign breast duct to cancer^32^. Single-cell chromatin analysis of mammary glands in developing mice has further refined the chromatin signatures associated with normal breast cellular lineages^33^. Beyond understanding chromatin accessibility in animal models^33–35^ and within immune cells^15^, there has been no investigation of the epigenetic state of human BCs across subtypes and their progenitors at a single-cell resolution.

Genome-wide expression profiling identified five biological BC subtypes^36^, namely luminal A/B, HER2-enriched, basal-like and normal breast-like, which differ by hormonal receptor status, proliferation, genomic instability, mutational signatures, treatment response and prognosis^37–41^. It has long been hypothesized that the high degree of heterogeneity in BC is due to different cells of origin within the breast duct. Evidence has mounted that the similarity between basal-like BC and basal/myoepithelial breast cells is superficial, whereas the cell of lineage for basal-like BC belongs to the luminal lineage. This paradigm was initially supported by early work on BRCA1-deficient mammary cells, where tumors arise from mammary basal cells^42^. Molyneux and colleagues analyzed a conditional mouse model of *BRCA1* deficiency that developed tumors resembling human basal-like BC and showed that these arose from a luminal ER-negative (ER^−^) progenitor population^43^. This is in line with evidence in humans, where *BRCA1* mutation carriers have been shown to harbor an expanded population of luminal progenitor cells with an aberrant phenotype, including expression of some basal epithelial cell markers^44,45^. Gene expression profiling from *BRCA1* heterozygous breast tissue showed similarities between luminal progenitor cells and basal-like breast tumors, and between luminal mature cells and luminal A/B tumors^44^. To address the cell of origin, Keller and colleagues isolated luminal (EPCAM^+^CD10^−^) and basal (CD10^+^) cells from *BRCA1* wild-type breast reduction specimens and upon implantation into immunodeficient mice, luminal cells gave rise to tumors resembling both luminal and basal-like subtypes, whereas basal cells gave rise to tumors not closely resembling either basal-like or luminal tumors^46^.

More recently, single-cell RNA sequencing (scRNA-seq) gene expression profiling has been used to establish links between breast tumor subtypes and benign duct cell types. Hu and colleagues performed scRNA-seq on breast tumors from *BRCA1* mutation carriers and noncarriers and found similarities between basal-like tumors and the expanded, abnormal luminal progenitor population seen in *BRCA1* carriers, and between ER-positive (ER^+^) breast tumors and luminal mature cells^8^. Additionally, scRNA-seq from fluorescence-activated cell sorting (FACS)-sorted luminal epithelial cells from reduction mammoplasties showed gene expression similarity between ductal KRT15^+^ luminal progenitors and published signatures of basal-like BC^47^. These studies have largely focused on protein markers and gene expression patterns. In this study, we apply single-nucleus RNA sequencing (snRNA-seq)/scRNA-seq and single-nucleus ATAC sequencing (snATAC-seq) in tandem to clarify not only the gene expression similarities between BC subtypes and their proposed cells of origin, but also the transcriptional networks responsible for transformation and cell lineage identity.

This study aims to understand tumor heterogeneity and its relation to BC lineage at a single-cell resolution. Chromatin accessibility, TF motif enrichment and their impact on the transcriptome reveal the structure of BC heterogeneity through integration of bulk-RNA/DNA sequencing, scRNA-seq, snRNA-seq and snATAC-seq technologies. Specifically, in this work we explore the transcriptional programs and chromatin accessibility patterns that link BC subtypes to distinct cell types in the benign breast duct. As part of the Washington University Human Tumor Atlas Network (WU-HTAN) program, we generated multi-omic data for 70 samples from 38 ER^+^PR^−^HER2^−^, ER^+^PR^+^HER2^−^, HER2^+^ and triple-negative BC (TNBC) tumors, 4 normal adjacent tissues and 1 metastatic liver sample. Of these patients, 27 were treatment-naive and 12 had undergone previous therapy. We observed subtype-specific chromatin accessibility features associated with driver gene expression signatures. We identified gene expression and chromatin accessibility networks shared between BC subtypes and benign breast duct populations at the single-cell level, which are mapped to specific structures by co-detection by indexing (CODEX) multiplex imaging. These findings may guide our understanding of the early pathogenesis of BC.

## Results

### Clinical features and genomic characterization

We conducted scRNA-seq and/or snRNA-seq for 57 tissue samples across 37 resected breast tumors (‘cases’) (Supplementary Table 1). Of these, 6, 16, 4 and 11 tumors were clinically annotated as ER^+^PR^−^HER2^−^, ER^+^PR^+^HER2^−^, HER2^+^ and TNBC, respectively. For a subset of tumors (*n* = 14), we collected up to three spatially distinct samples from the same tumor using our grid processing method for sample collection (Fig. 1b,c). In addition, each sample also underwent extensive imaging characterization and bulk omics. The data generated included scRNA-seq, snRNA-seq, snATAC-seq, spatial transcriptomics (ST), bulk whole-exome sequencing (WES) and bulk-RNA sequencing (bulk-RNA-seq). We generated scRNA data for 31 samples (from 14 cases), snRNA data for 30 samples (from 27 cases) and snATAC data for 25 samples (from 24 cases), of which 4 samples had both scRNA and snRNA for comparison. Additional validation was provided from ST data comprising 33 slides from 13 BC cases and CODEX multiplex imaging on 47 slides from 13 cases. Overall, 54 and 52 paired samples underwent bulk WES and RNA-seq, respectively.

Of 37 patients with resected breast tumors, samples were obtained from 26 patients before treatment and 11 patients following therapy (Supplementary Table 1). Systemic treatment regimens for previously treated patients included carboplatin and paclitaxel; doxorubicin and cyclophosphamide followed by paclitaxel; paclitaxel, trastuzumab and pertuzumab; single-agent paclitaxel; doxorubicin, cyclophosphamide and pembrolizumab; and aromatase inhibitors. Two patients who had not yet received treatment for the breast tumor included in this study had previously received treatment for previous unrelated BC. The median age of patients was 61. Three patients under the age of 40 were included in this cohort (ages 30, 31 and 38) (Fig. 1d and Supplementary Table 1). Of these, the patient aged 30 (HT163B1) had a family history, including two other family members with BC diagnosed in their thirties. In all, 21 of the 37 patients had a known first-degree family member with a cancer diagnosis, though only 7 of these were known to be BCs. The majority of tumors (30 of 37) were histologically identified as invasive ductal carcinoma and the other 7 of 37 were invasive lobular carcinoma (Supplementary Table 1).

We determined somatic and germline variants in the cohort (Fig. 1e and Supplementary Tables 2 and 3) using WES. Consistent with previous studies, we detected several cases with somatic mutations in *TP53* and *PIK3CA* (Fig. 1e). For germline variants, we identified two potential pathogenic germline variants in *BRCA2* (p.A938fs in HT243B1 and p.K2013* in HT271B1) and one in *BRIP1* (p.K703fs in HT077B1) using the CharGer pipeline^48^. Notably, these predicted pathogenic germline variants seem to be present at a much higher variant allele fraction (VAF) in the tumor samples compared to paired normals for the affected cases, showing significant loss of heterozygosity (one-sided Fisher exact test adjusted *P* values (false discovery rate, FDR), 4.60 × 10^−5^ and 4.18 × 10^−5^ for the *BRCA2* variants and 1.98 × 10^−14^ for the *BRIP1* variant). One 30-year-old patient with a family history of BC (HT163B1) has a germline frameshift variant of unknown significance (p.Y1672fs) in *BRCA2* that has significant loss of heterozygosity in the tumor (FDR = 0.0001). Across spatially separate samples from the same case, we generally detected the same somatic mutations across samples, with a few exceptions likely due to tumor purity. The two cases with somatic mutations in *CDH1* were of lobular histology.

**Extended Data Fig. 1:**
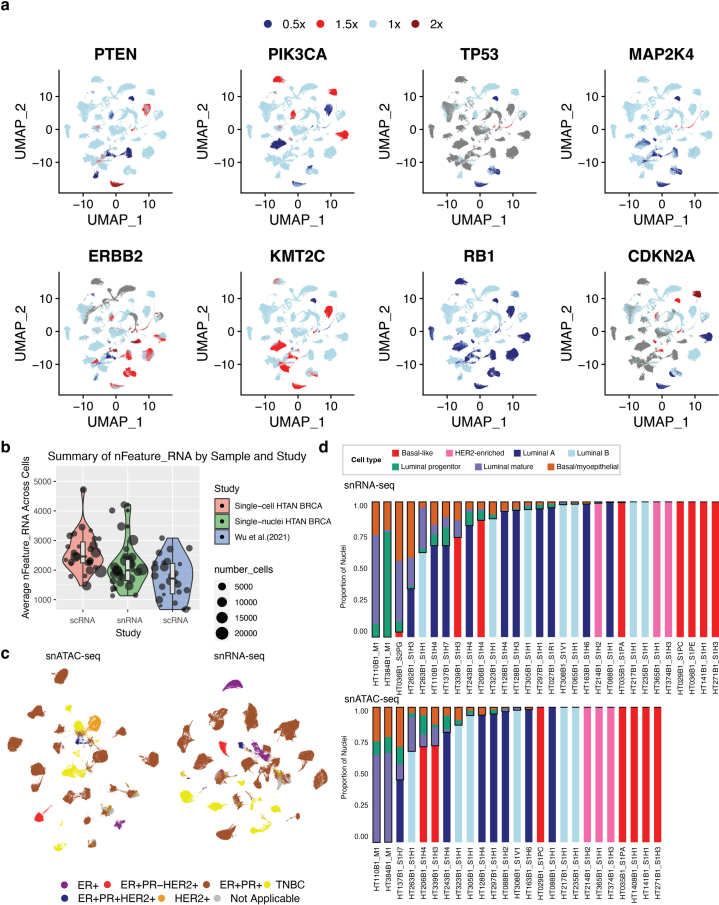
InferCNV, nFeature count and epithelial cell type distribution. **a**) UMAP plots of copy number events from inferCNV mapped to epithelial cells derived from snRNA data. **b**) Violin plot of the average nFeature_RNA detected across each sample across three cohorts (one external dataset Wu et al. 2021 and two internal HTAN cohorts). Size of dots indicate the number of cells detected for each sample and box-plot is overlaid on violin plot (scRNA HTAN BRCA n = 31 samples (from 14 cases), snRNA HTAN BRCA n = 30 samples (from 27 cases), scRNA Wu et al. n = 26 samples). The boxplots show the median with 1.5 × interquartile range whiskers. **c**) UMAP representations of epithelial subsets for snATAC and snRNA samples colored by clinical subtype. **d**) (Left) Barplots indicating proportion of epithelial nuclei per sample identified for the snRNA-seq data. (Right) Barplots indicating proportion of epithelial nuclei per sample identified for the snATAC-seq data. Source data

After filtering and quality control (QC), we obtained a total of 138,835 cells and 205,120 nuclei, which we clustered and classified into cell types based on marker gene expression (Methods). For cases with paired WES, we identified copy number alterations that overlap InferCNV calls derived from the single-cell data to confidently identify tumor subpopulations relative to normal cells (Extended Data Fig. 1a and Supplementary Table 4). In addition to tumor cells, we identified stromal cells of the breast, including endothelial cells, cancer-associated fibroblasts of the vascular (vCAF), matrix (mCAF), developmental (dCAF) and cycling (cCAF) subsets and adipocytes. Within the benign breast compartment, we captured benign duct cells, including luminal mature cells, luminal progenitor cells and basal/myoepithelial cells. Lymphocyte subsets include B cells, plasma cells and CD4^+^ or CD8^+^ T cells, with T cells being further subdivided, including regulatory T (T_reg_), cytotoxic, pre-exhausted, exhausted, activated and proliferating cell subsets. Other immune components including monocytes, macrophages, dendritic cells including classical (cDC1 and cDC2) and plasmacytoid (pDC), natural killer (NK) cells, NKT cells and mast cells were also identified (Fig. 1f–h). We calculated the fraction of tumor cells for samples with adequate coverage ranging from 1.6% to 82% in scRNA and 1.5% to 99% in snRNA. Related to other work, this study provides high-quality single-cell data (snRNA, mean 2,187 genes per cell; scRNA, mean 2,448 genes per cell) relative to other large cohorts for BC^10^ (Extended Data Fig. 1b). As previously reported, while snRNA-seq and scRNA-seq both capture similar cell type composition in each assay, the proportions can vary dramatically with frozen tissue nuclei isolation techniques (snRNA) capturing a higher tumor fraction and fresh tissue whole cell (scRNA) dissociation capturing more immune cells^49,50^. To take advantage of these differences, we explored tumor heterogeneity using snRNA-seq/snATAC-seq and the tumor microenvironment using scRNA-seq. Further, for some cases with paired scRNA-seq and snRNA-seq data (from different regions of the same tumor), we validated findings using the orthogonal method. In summary, we generated a large compendium of single-cell data encompassing both RNA (snRNA-seq and scRNA-seq) and ATAC data spanning three subtypes of BC and normal adjacent tissues to study tumor heterogeneity and normal to tumor transition states.

### Tumor subtype intrinsic and extrinsic characterization

Historically, breast tumor subtype assignments were calculated from bulk-RNA-seq data using published methods for the PAM50 assay^51^; however, assigning tumor subtypes from gene expression is confounded by the composition of tumor and non-tumor cells in a sample. To disentangle subtype assignment from stromal contribution, the PAM50 algorithm was applied separately to bulk-RNA-seq and snRNA-seq data (Methods). Subtype assignments from bulk-RNA-seq and snRNA-seq demonstrate good concordance: 12 of 14 samples (85%) with both bulk-RNA-seq and snRNA-seq had identical PAM50 calls from both modalities. Of the discrepant cases, the bulk-RNA-seq-based assignments were normal-like and luminal A, and both cases were called luminal B from snRNA-seq. PAM50 subtype assignments from our cohort (Supplementary Table 1) also closely mirrored clinical biomarker profiles. Thirteen of 15 TNBC samples with bulk-RNA-seq or snRNA-seq data (87%) were assigned to the basal-like subtype, with two assigned as HER2-enriched (Fig. 1d and Supplementary Table 1). Sixteen of 38 (42%) clinically defined as ER^+^HER2^−^ samples (with or without PR positivity) with bulk-RNA-seq or snRNA-seq data were assigned to the luminal A subtype, with another 19 (50%) assigned to the luminal B subtype. The remaining four (8%) ER^+^HER2^−^ samples were assigned to the basal-like subtype. Three clinical HER2^+^ samples were included in this dataset and all were classified as HER2-enriched by PAM50.

**Fig. 2:**
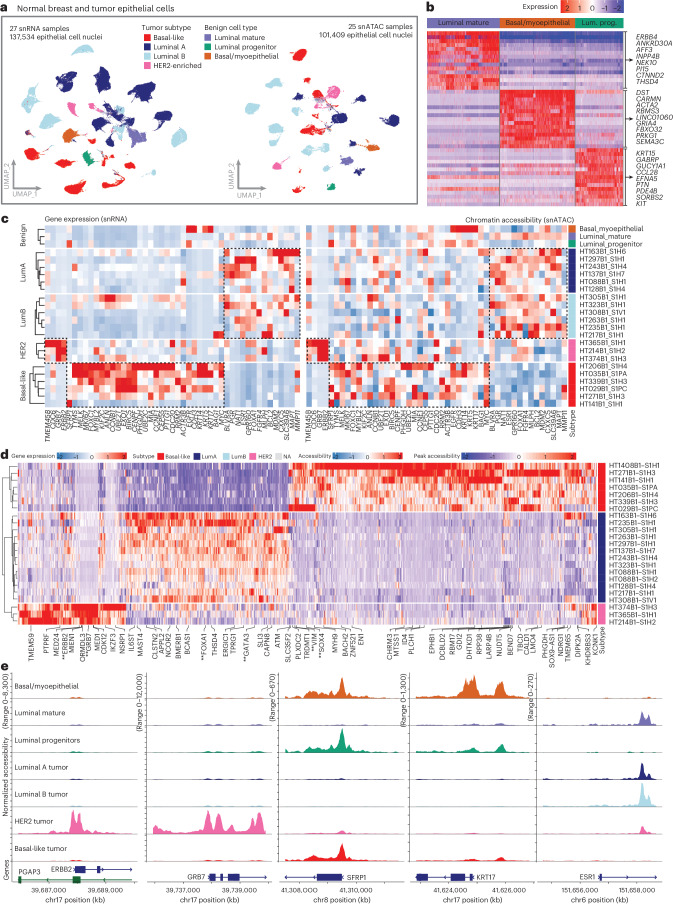
Tumor subtype and benign duct cell types. **a**, UMAP plots of benign breast epithelial cells and BC cells for all snRNA (left) and snATAC (right) samples. Tumor cells colored by PAM50 subtype. **b**, Heatmap of top 15 DEGs in snRNA-seq data from benign breast duct cells. A subset of genes from each benign cell type is highlighted in the figure. **c**, Heatmaps of snRNA gene expression (left) and snATAC chromatin accessibility (right) for genes in the PAM50 subtyping assay. Average values are shown for all tumor cells per sample, as well as each benign breast duct cell type pooled across samples (top). Characteristic genes identifying luminal A/B, HER2-enriched and basal-like subtypes are shown in boxes. **d**, Peak accessibility for differentially accessible promoters by BC subtype in snATAC-seq data. Key subtype-associated genes are highlighted in bold and with two asterisks below. **e**, Coverage plots showing normalized chromatin accessibility across promoter regions of key subtype-associated genes in snATAC-seq data from tumor nuclei grouped by subtype and benign epithelial cell types.

Just as average expression of PAM50 genes in tumor cells from snRNA-seq could discriminate tumor subtype, chromatin accessibility in the promoters of PAM50 genes from snATAC-seq showed good segregation of tumors by subtype in the 21 samples (from 21 cases) with both data types available (Fig. 2a–c). In addition to BC cells, benign epithelial ductal cells were identified and stratified into three benign cell types, using both published expression markers and co-clustering status across samples^5,6,8^. Each benign cell type harbored unique markers, namely *KIT*, *KRT15* and *PTPN* for luminal progenitor (LP) cells, *ANKRD30A*, *ERBB4*, and *AFF3* in luminal mature (LM) and *ACTA2*, *RBMS3* and *DST* in basal/myoepithelial progenitor (BP) cells. We did not identify a robust population of mammary stem cells, consistent with their low abundance in adults^3^. Benign ductal cells were detected in all clinical subtypes (ER^+^, ER^+^/PR^+^, HER2^+^ and TNBC) and PAM50 subtypes (luminal A, luminal B, HER2-enriched and basal-like) (Extended Data Fig. 1c). Across all samples, we identified all three progenitor cell types in 46% (*n* = 24) and 36% (*n* = 14) of samples in the scRNA and snRNA cohorts, respectively (Extended Data Fig. 1d). Compared to other benign ductal cells, LM cells expressed high levels of *ERBB4*, *DACH1 and ESR1*, with hormone-response pathways enriched among differentially expressed genes (Fig. 2b and Supplementary Table 5). In contrast, LP cells were characterized by high *KIT* expression as well as expression of other progenitor markers (*ALDH1A3*). Finally, BP cells showed high expression of genes involved in cytoskeleton and myoepithelial contraction, including *ACTA2* and *DST*, as well as *TP63*. (Fig. 2b). Genes included in the PAM50 subtyping assay show dramatic differences by subtype even by snRNA measurements and are further confirmed in the snATAC data (Fig. 2c). Differentially accessible promoters by subtype highlighted key subtype-associated genes including *VIM* and *SOX4* in basal-like tumors, *FOXA1* and *GATA3* in luminal tumors and *ERBB2* and *GRB7* in HER2-enriched tumors (Fig. 2d). Promoter accessibility of PAM50 genes showed stark subtype differences and highlighted similarities to benign duct populations (Fig. 2e). The key basal-like genes *SFRP1* and *KRT17* showed high promoter accessibility in basal-like tumors and LP cells, whereas the key luminal gene *ESR1* showed promoter accessibility in luminal A/B tumors and LM cells. By analyzing over 20 samples from various patients, we have built a large resource of both BC cells and benign duct populations, enabling us to evaluate the transcriptional programming responsible for the normal to tumor cell transition across multiple subtypes of BC.

**Fig. 3:**
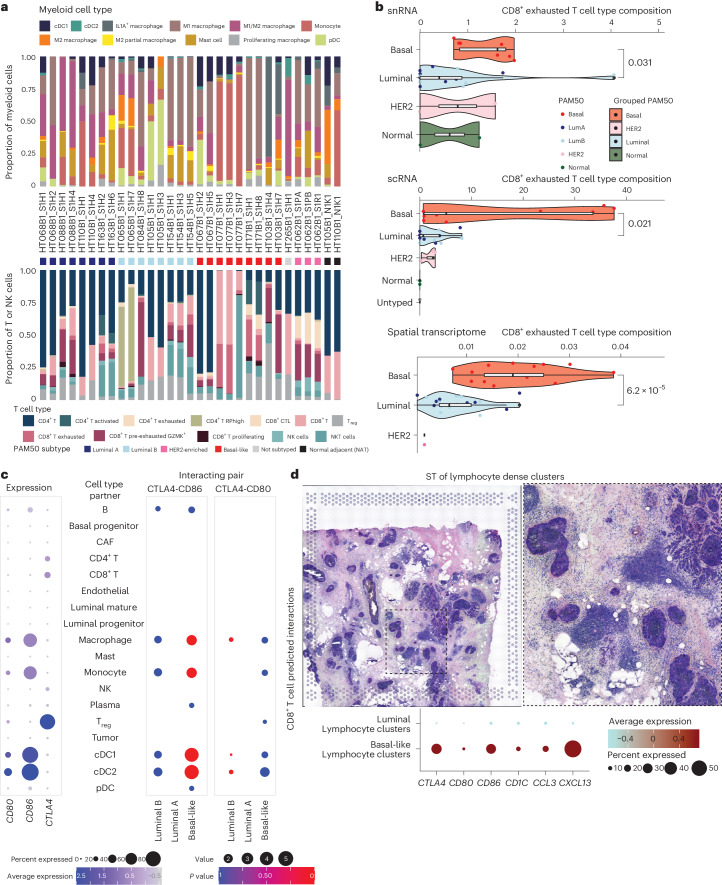
Subtype-enriched elements of the tumor microenvironment. **a**, Composition of myeloid immune subsets (top) and T/NK subsets (bottom) for each sample with scRNA-seq data. **b**, Proportion of Exhausted CD8^+^ T cells by subtype identified by snRNA-seq, scRNA-seq and ST. Each dot is the proportion of exhausted CD8^+^ T cells relative to other T cells for an individual piece for the snRNA and scRNA, whereas for the ST it is based on the proportion of total spots. The box-plots show the median with 1.5 × interquartile range whiskers. scRNA (basal, 9 samples, 4 cases; luminal, 16 samples, 8 cases; HER2, 3 samples, 1 case; normal, 2 samples, 2 cases; untyped, 1 sample, 1 case); snRNA (basal, 7 samples, 7 cases; luminal, 14 samples, 14 cases; HER2, 2 samples, 2 cases; normal, 2 samples, 2 cases); ST (basal,13 sections, 4 cases; luminal, 19 sections, 8 cases; HER2, 1 section, 1 case). A Wilcoxon test (default, two-sided) was used for all comparisons. **c**, Expression of three markers (*CD80*, *CD86* and *CTLA4*) in the RNA (left). The size of the dot indicates the percentage of genes expressing the gene and the color indicates average expression. CellPhoneDB results indicating interacting gene partners in the scRNA-seq data (right). Size of dot indicates mean expression of interacting gene partners in their respective cell types and color indicates *P* value. **d**, Example of a lymphocyte-dense region in one sample of interest (top). A zoomed-in region of the left image, which we use to quantify the expression of various markers in the bottom panel (right). Expression of a subset of genes in lymphocyte-dense clusters isolated from ST data from luminal and basal cancers. The size of the dot indicates the percent of the spots included in the analysis that express the gene of interest and the color indicates average expression. Source data

**Extended Data Fig. 2:**
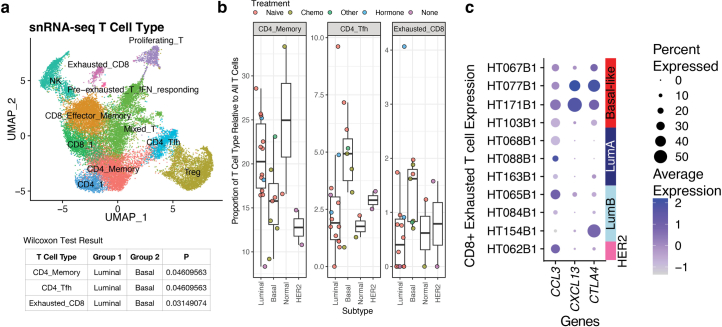
Exhausted CD8 T cell analysis in snRNA-seq data. **a**) UMAP of T cells identified in snRNA-seq data. Cells are colored by cell types. **b**) Boxplots show the proportion of T cell types relative to all T cells for each piece of tissue separated by subtype and by T cell type. Each point is colored by Treatment Status. The boxplots show the median with 1.5 × interquartile range whiskers. Sample numbers for the box-plot include the following CD4 memory (Luminal: 14 samples, 4 cases; Basal: 7 samples, 7 cases; Normal: 2 samples, 2 cases; HER2: 2 samples, 2 cases), CD4_Tfh(Luminal: 14 samples, 14 cases; Basal: 7 samples, 7 cases; Normal: 2 samples, 2 cases; HER2: 2 samples, 2 cases), Exhausted_CD8 (Luminal: 14 samples, 14 cases; Basal: 7 samples, 7 cases; Normal: 2 samples, 2 cases; HER2: 2 samples, 2 cases).Table labeled Wilcoxon test result shows the P value associated with the comparison of proportions of T cells between Group 1 and Group 2. **c**) Plots showing expression of CCL3, CTLA4, and CXCL13 in Exhausted CD8+ T cells. Size of dot indicates % of cells expressing the gene of interest while color indicates average expression. Source data

**Extended Data Fig. 3:**
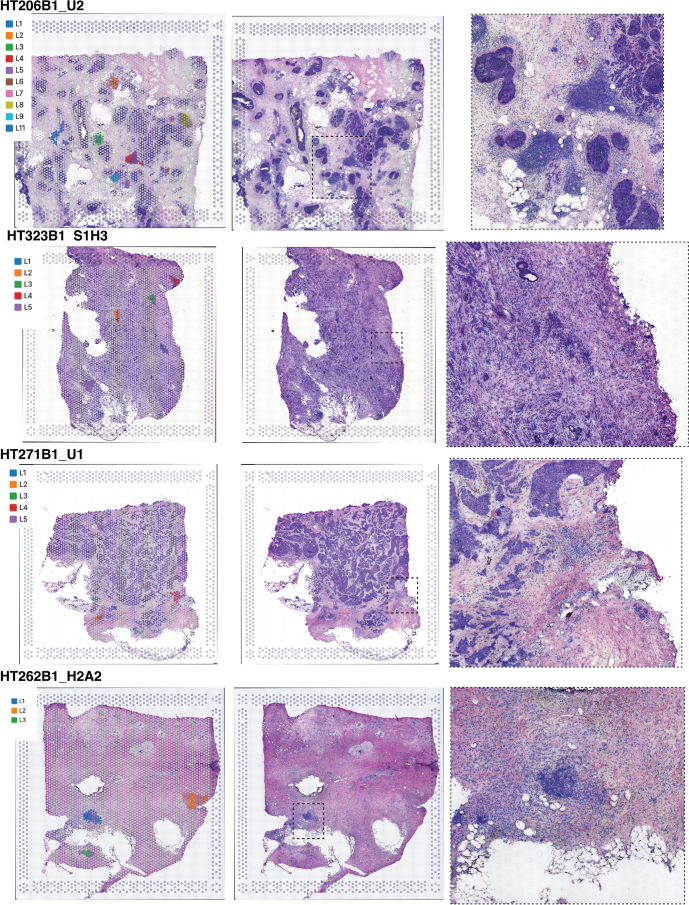
Lymphocyte dense regions in spatial transcriptomics data. Each row indicates a section of a different sample. Left image indicates the lymphocyte dense clusters (L1-LX) selected for evaluating gene expression differences between subtypes. Middle image is the H&E with a region indicated in dashed box that is zoomed in on the right plot to show how we identified lymphocyte dense regions for our analysis.

**Extended Data Fig. 4:**
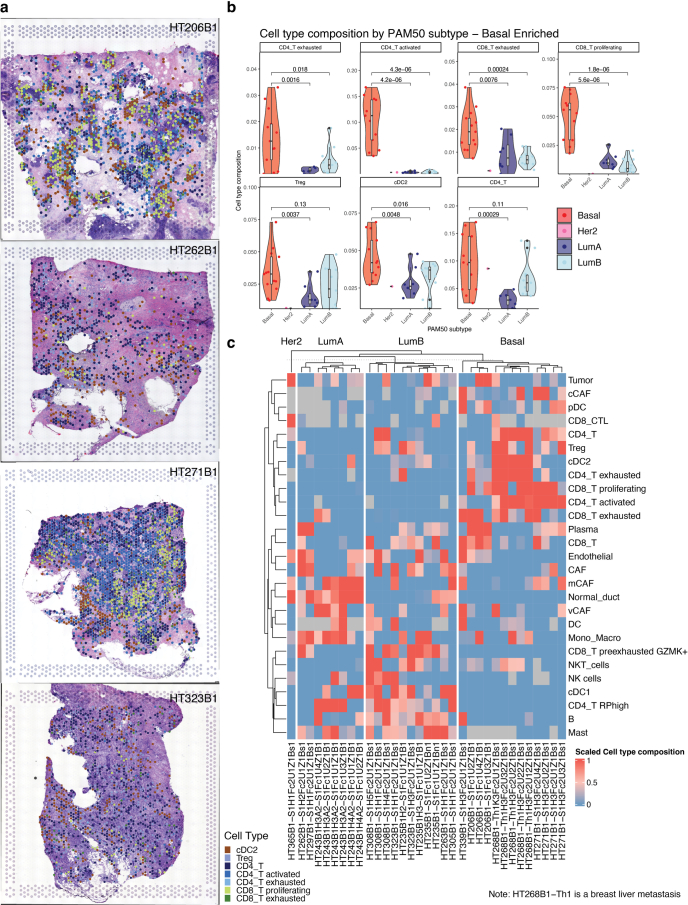
Spatial mapping of snRNA-seq cell types to Spatial Transcriptomics Data using CytoSPACE. **a**) CytoSPACE mapping results of CD4, CD8, Treg and cDC2 to a subset of luminal and basal samples. **b**) Violin plots of cell type composition of basal enriched cell types. Each grouped violin is separated by cell type and subtype. P-values are derived from stat_compare_means using the method = t.test. The boxplots show the median with 1.5 × interquartile range whiskers. For all box-plot sample numbers are as follows: Luminal 19 samples, 8 cases; Basal 13 samples, 4 cases; Her2 1 samples, 1 case. **c**) The heatmap represents the scaled cell type proportion across all breast spatial transcriptomic samples. Source data

In addition to profiling tumor and benign ductal cells, we also examined subtype differences in the immune compartment. Lymphoid and myeloid cells were profiled in 31 scRNA-seq samples comprising 29 tumor samples and two normal adjacent tissues samples (Fig. 3a). Exhausted CD8^+^ T cells were significantly more prevalent in basal-like tumors compared to luminal A or B tumors (Fig. 3a,b). This finding was consistent in snRNA-seq data and independent of treatment (Fig. 3b and Extended Data Fig. 2a,b). We performed cell–cell interaction analysis using CellPhoneDB and observed significant predicted interactions between *CTLA4* expressed by CD8^+^ T cells and CD86 expressed by multiple myeloid cell types (macrophages, monocytes, cDC1 and cDC2) in basal-like samples relative to luminal samples (Fig. 3c) (Methods)^52^. *CTLA4* on CD8^+^ T cells was also predicted to interact with *CD80* on various myeloid cell types in basal-like tumors, though this did not reach statistical significance. Compared to luminal A or B tumors, exhausted CD8^+^ T cells in basal-like tumors expressed higher levels of *CTLA4*, *CXCL13* and *CCL3* (Extended Data Fig. 2c). To validate this finding, we utilized ST data from the 10x Visium platform. For two basal (HT206B1 and HT271B1) and two luminal (HT323B1 and HT262B1) samples, the ST spots overlapping lymphocyte-dense regions were extracted (Fig. 3d and Extended Data Fig. 3). We hypothesized that if cell–cell interactions between myeloid and T cell populations maintained differential interactions between subtypes as shown in the single-nuclei data, then this would hold true for each subtype in lymphocyte-dense regions derived from ST data. The ST data confirmed that *CTLA4*, *CD80*, *CD86* and *CD1C* had an overall higher expression across two basal samples relative to the two luminal samples (Fig. 3d). Finally, we performed cell type deconvolution using CytoSPACE on 33 slides from four basal-like, eight luminal and one HER2 BCs and again observed increased abundance of exhausted CD8^+^ T cells in basal-like cancers (Fig. 3b and Extended Data Fig. 4)^53^.Taken together, this provides evidence of increased immunosuppression and exhaustion in T cells in basal-like breast tumors.

### Cell of origin and regulons of putative tumor lineages

**Fig. 4:**
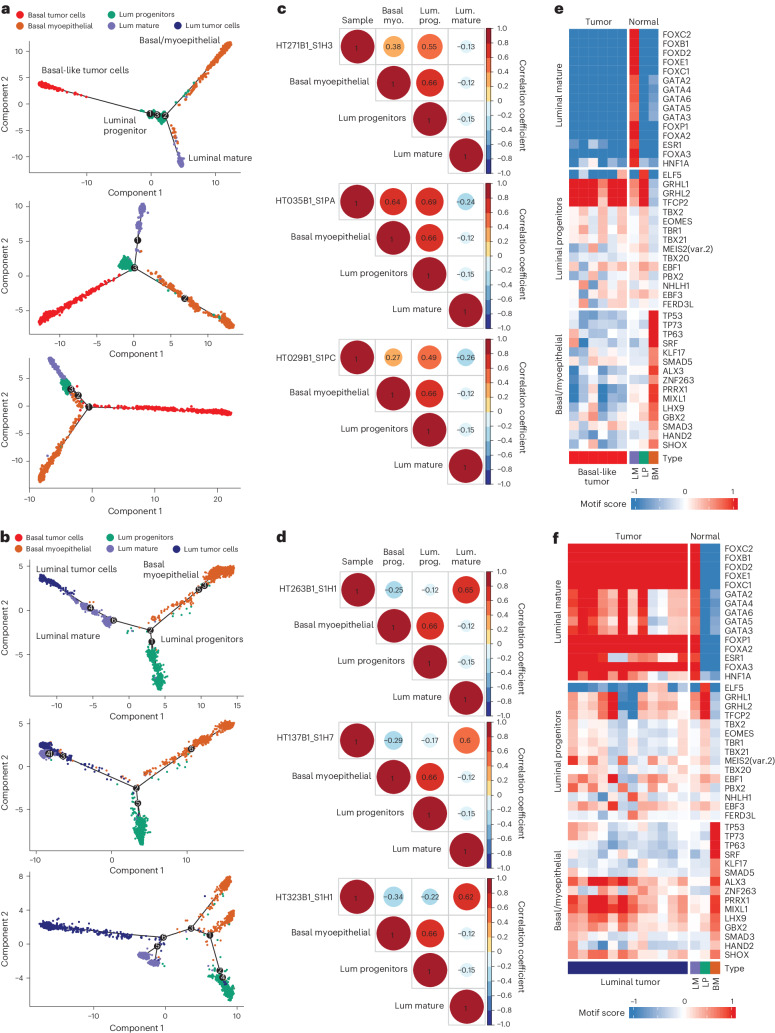
Chromatin accessibility evidence for subtype-specific cell of origin. **a**, Monocle pseudotime plots of tumor and benign breast duct cells from three representative basal-like BC samples. **b**, Monocle pseudotime plots of tumor and benign breast duct cells from three representative luminal BC samples. **c**, Correlation matrices for TF motif scores from tumor cells and benign duct cells for the BC samples in **a**. **d**, Correlation matrices for TF motif scores from tumor cells and benign duct cells for the BC samples in **b**. **e**, Heatmap of motif scores for the top 15 differentially accessible motifs identified in LM, LP and BP cells. Scores are shown for tumor cells from each basal-like snATAC-seq sample and for benign breast duct cells. **f**, Heatmap of motif scores for the top 15 differentially accessible motifs identified in LM, LP and BP cells. Scores are shown for tumor cells from each luminal snATAC-seq sample and for benign breast duct cells.

**Extended Data Fig. 5:**
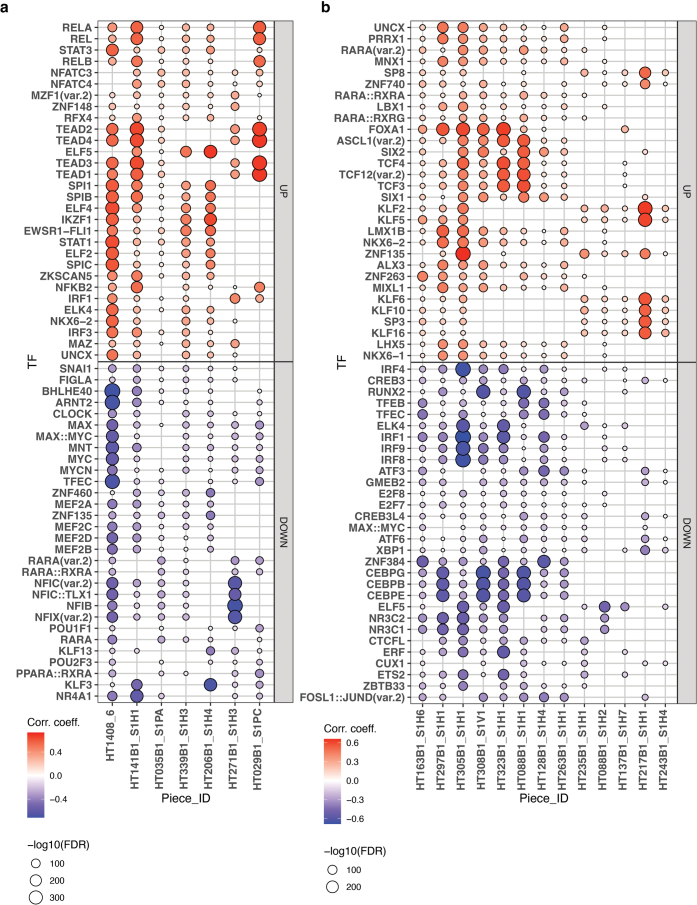
TF motifs and pseudotime correlation. **a**) Correlation of TF motifs’ scores with pseudotime from precursors to tumor cells from basal-like samples. Color of dot indicates correlation coefficient of each TF separated by sample while the size relates to significance (by FDR). **b**) Correlation of TF motifs’ scores with pseudotime from precursors to tumor cells from luminal samples.

Identifying and understanding the cells that give rise to BC is critical for comparing tumor and normal cells and ultimately for understanding tumor progression and evolution. While there is no consensus in the field of which precise cell types give rise to tumor cells in BC, the prevailing model is that LP cells tend to give rise to TNBC cancers, whereas LM cells develop into ER^+^ or ER^+^PR^+^ tumors^3,46^. The chromatin landscape at single-cell resolution is uniquely suited to reconstruct the lineage between progenitor populations and malignant cells in a tumor. To determine whether tumor subtypes were associated with distinct cells of origin, we performed Monocle trajectory analysis on snATAC-seq data from basal-like and luminal A/B tumor cells and benign LM, LP and BP cell populations (Methods). We observed for the majority of basal-like cases that tumor cells were closely associated with LP cell populations, whereas for the majority of the luminal cases, we observed tumor cells to be closer to LM cells (Fig. 4a,b). Correlation of motif scores across epithelial cell types in individual cases also highlighted greater similarity between basal-like BC and LP cells and between luminal BC and LM cells (Fig. 4c,d). Finally, motifs showing high chromatin accessibility in LP cells were also highly represented in open chromatin in basal-like breast tumors (Fig. 4e), whereas motifs found in LM cells were also highly represented in open chromatin in luminal breast tumors (Fig. 4f). Motifs that exhibited differential accessibility in LM cells and were also enriched in open chromatin in luminal tumors included forkhead family proteins, GATA3, ESR1 and HNF1A. Differentially accessible motifs for LP cells also enriched in open chromatin in basal-like tumors included GRHL1 and TFCP2. The TFs for which accessibility was correlated with pseudotime between luminal tumors and LM cells, and between basal-like tumors and LP cells, are shown in Extended Data Fig. 5.

**Extended Data Fig. 6:**
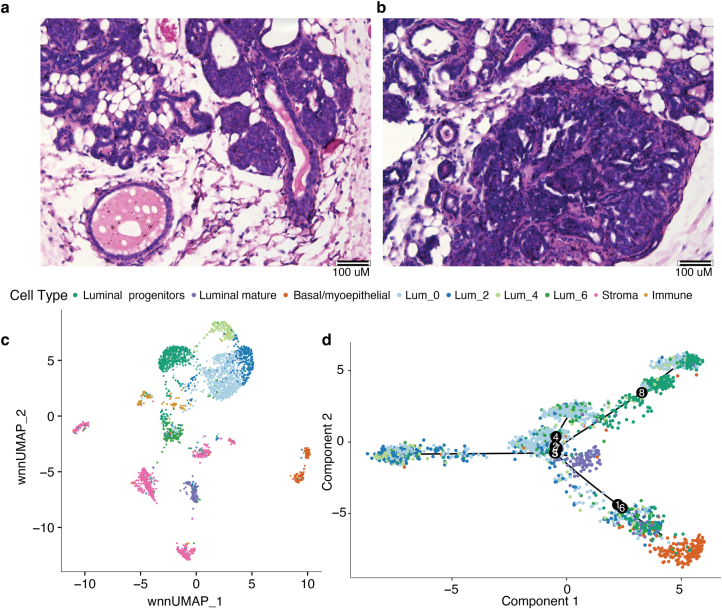
Histology and snATAC data from MMTV-PyMT model. **a**) H&E mouse mammary glands at 12 weeks indicating normal ducts and cancer cells. One replicate indicated in figure. **b**) A second H&E of mouse mammary glands at 12 weeks indicating normal ducts and cancer cells. One replicated indicated in figure. **c**) UMAP of single-nucleus ATAC-seq data from mouse model. Points are colored by cell type. **d**) Monocle trajectory analysis of epithelial derived cells from snATAC-seq data. Each point is colored by cell type.

To explore precancer states during early malignancy, we evaluated tumor and normal cells in the MMTV-PyVT mouse model of luminal BC^54^. Mouse mammary glands were collected at 12 weeks old to capture the transition of normal ducts to cancer cells. Both normal ducts and cancer cells were recognized in the hematoxylin and eosin (H&E) staining of embedded samples (Extended Data Fig. 6a,b). This is concordant with snATAC data derived at the same time point showing that both early stages of cancer cells and normal ducts are present in the mouse model. Trajectory analysis using Monocle on the ATAC data again confirmed the transition of proposed cancer cells (Lum_0, Lum_2, Lum_4 and Lum_6) from LM cells rather than progenitors or basal/myoepithelial cells (Extended Data Fig. 6c,d).

**Fig. 5:**
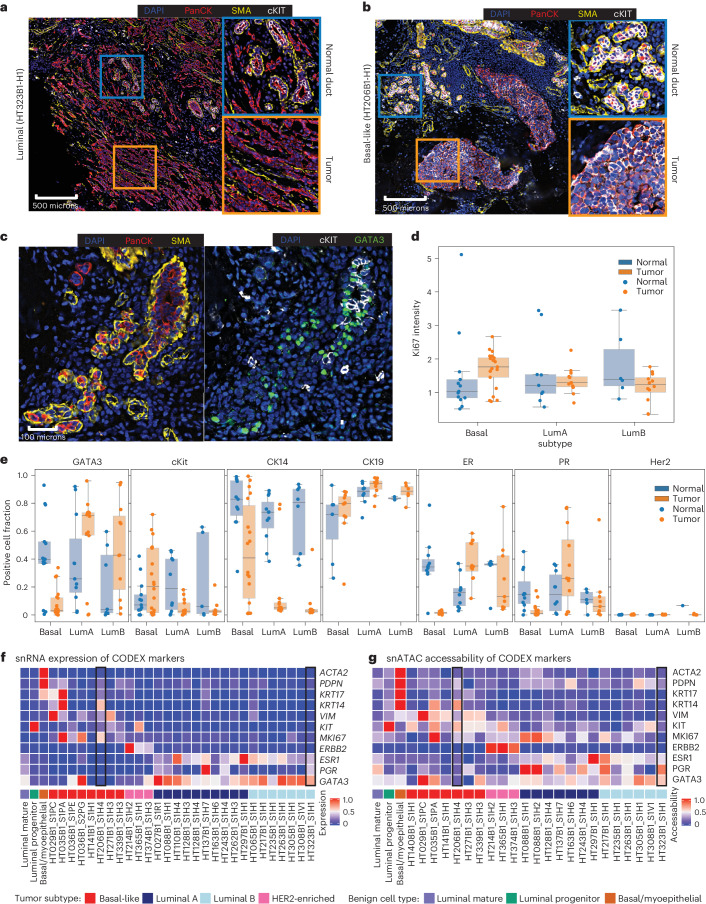
Spatial characterization of tumor subtype and normal ducts. **a**, CODEX multiplex immunofluorescence on luminal sample HT323B1. Inset regions (square) are expanded to the right and colored by related inset. DAPI is stained in blue, PanCK in red, SMA in yellow and c-KIT in white. One replicate indicated in figure. **b**, CODEX multiplex immunofluorescence on basal sample HT206B1. Inset regions (squares) are expanded to the right and colored by related inset. DAPI is stained in blue, PanCK in red, SMA in yellow and c-KIT in white. One replicate is indicated. **c**, Section of CODEX immunofluorescence image from HT206B1 centered on a benign ductal region. Section on the left is stained with DAPI in blue, PanCK in red and SMA in yellow. The section on the right is stained with DAPI in blue, c-KIT in white and GATA3 in green. One replicate is indicated. **d**, Box-plot summarizing overall Ki67 intensity across all samples (49 sections and 21 samples) in normal duct and tumor regions separated by subtype. The box-plots show the median with 1.5 × interquartile range whiskers. **e**, Positive cell fraction of GATA3 (45 sections and 19 samples), c-Kit (42 sections and 17 samples), CD14 (44 sections and 20 samples), CK19 (27 sections and 8 samples), ER (39 sections and 14 samples), PR (39 sections and 14 samples) and Her2 (33 sections and 9 samples) across all samples in normal duct and tumor regions separated by subtype. **f**, Average expression scores of CODEX marker genes in the snRNA-seq data. Gene expression for samples HT206B1_S1H1 and HT323B1_S1H1 used for CODEX imaging are outlined. The box-plots show the median with 1.5 × interquartile range whiskers. **g**, Average chromatin accessibility scores of CODEX marker genes in snATAC-seq data. Chromatin accessibility for samples HT206B1_S1H1 and HT323B1_S1H1 used for CODEX imaging are outlined. Source data

To confirm markers for single-cell/single-nucleus populations and provide support for benign cell types and their connection to putative cell of origin, we performed CODEX multiplex imaging on representative basal-like and luminal BC sections. SMA, podoplanin and vimentin were used for staining basal myoepithelial cells, and c-Kit and GATA3 were used for LP cells and LM cells, respectively (Methods). As a result, tumor cells from the luminal B tumor HT323B1 exhibit tumor cells positive for ER and PR, with a lower proliferative signature by Ki67 staining and a lack of c-Kit staining (Fig. 5a). Tumor cells from basal-like tumors had a strong proliferative Ki67 staining and high c-Kit positivity (Fig. 5b). From the benign structures, basal myoepithelial cells are positive for SMA, podoplanin and vimentin, LM cells are positive for GATA3 and LP cells are positive for c-Kit (Fig. 5c). Notably, normal duct cells coexpressing c-Kit and GATA3 were rare and may have been due to cell segmentation errors. Quantification of immunofluorescence signal in areas of tumor and normal duct showed higher c-KIT positivity in normal duct compared to luminal tumor, but higher c-KIT positivity in basal tumor compared to normal duct (Fig. 5e). The increased c-KIT positivity in both basal tumor regions and normal LP cells further emphasizes the connection between these two cells from a proteomic view. Epithelial cell markers in normal duct and tumor populations in basal-like and luminal tumors are shown in Fig. 5e. Similarly, GATA3 showed increased positivity in normal duct and luminal tumor and decreased positivity in basal tumors (Fig. 5e). Additional epithelial markers were quantitated in tumor and normal duct regions with expected results, including increased CK14 positivity in basal tumors and increased ER and PR positivity in luminal tumors (Fig. 5e). Gene expression and chromatin accessibility for CODEX marker genes in snRNA-seq and snATAC-seq are consistent with these results (Fig. 5f–g).

**Fig. 6:**
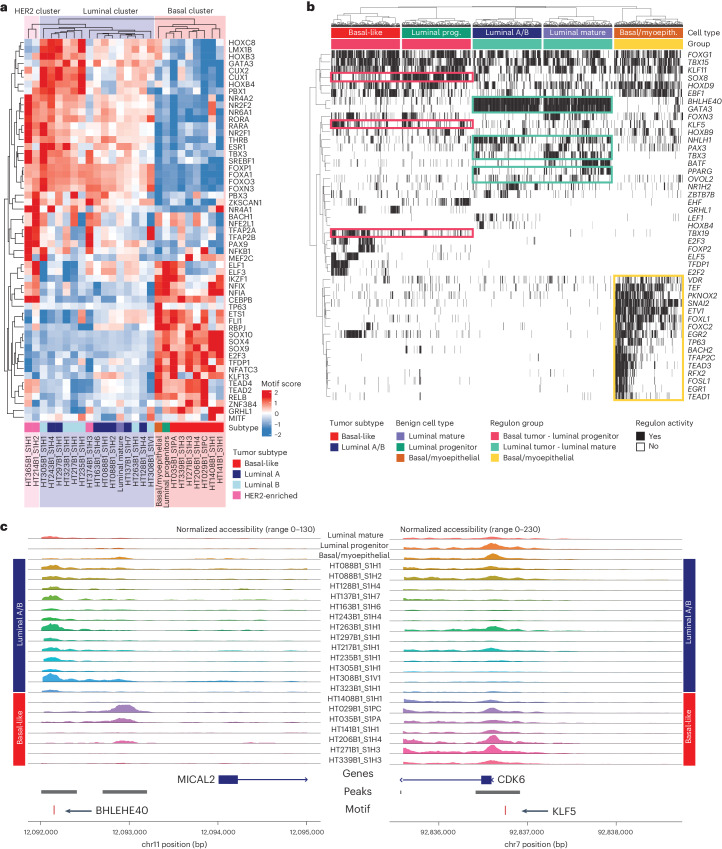
Tumor lineage-specific regulators of gene expression. **a**, Heatmap of differentially accessible motifs identified in tumor cell snATAC-seq data. Motif scores are shown for average value across tumor cells in each sample and for LP, LM and basal/myoepithelial cells pooled across all samples. **b**, Binarized heatmap of regulon activity in tumor-normal lineage groups. Color bars above show tumor/benign cell type and regulon group (basal-like BC and LP, luminal A/B BC and LM and basal myoepithelial). **c**, Coverage plots of normalized snATAC-seq accessibility across promoter regions for *MICAL2* (left) and *CDK6* (right). Regulon TF motifs and ATAC peak regions are shown below.

To explore the tumor cell of origin more deeply, we sought to reconstruct transcriptional networks specific to these distinct lineages. Grouping differentially accessible motifs of tumor cells from snATAC-seq, a high degree of similarity between LP cells and basal-like tumor cells and between LM cells and luminal A/B tumor cells was observed, whereas basal myoepithelial cells were distinct from all tumors (Fig. 6a). Key TF motifs enriched in the open chromatin of basal-like tumors and LP cells include NFIB, TEAD family TFs, SOX family TFs and CEBPB. In contrast, luminal A/B tumor cells and LM cells showed high accessibility for the ER ESR1, as well as forkhead proteins, including FOXA2 and FOXP1, GATA3 and other GATA-box TFs, and HNF1A. Two HER2-enriched samples had snATAC-seq data and showed enrichment for RARA, NR6A1 and ESRRA motifs. Basal myoepithelial cells showed high motif accessibility for TFs, including TP63, ZBTB18 and SRF.

To interrogate the genes linking tumor cells and their proposed cell of origin, we next used SCENIC to identify regulons of co-regulated genes in snRNA-seq data (Methods)^55^. Based on the evidence from motif accessibility and pseudotemporal association (Fig. 4), regulons were identified for three related lineage groups: (1) basal-like tumor and LP cells; (2) luminal tumors and LM cells; and (3) basal myoepithelial cells. Lineage-specific regulons for lineage group 1 include SOX8, KLF5 and TBX19 (Fig. 6b). In contrast, lineage-specific regulons identified for group 2 include BHLHE40, GATA3, NHLH1, PAX3, TBX3, OVOL2 and PPARG. Finally, regulons specific to basal myoepithelial cells include VDR, SNAI2, ETV1 and TP53. Of note, while *SOX8* expression has been implicated in TNBCs as a regulator of stem-like capabilities in tumor cells, its expression in LP cells has not been described^56^. Within lineage group 2 (luminal tumors and LM cells), the co-regulation of *BHLHE40* and *GATA3* are likely the result of hypomethylation in luminal A tumors^57^, which is also shared by LM cells in our dataset.

To provide orthogonal evidence for the importance of these TFs, we sought genes in these lineage-specific regulons with the regulon TF motif located in a differentially accessible ATAC peak in the gene’s promoter. Examples of this include the BHLHE40 motif in the promoter region of *MICAL2* and the KLF5 motif in the promoter region of *CDK6* (Fig. 6c). Within our sample cohort, the BHLHE40 motif upstream of *MICAL2* is accessible in LM and luminal A/B tumor cells and is less accessible in basal-like tumor samples. BHLHE40 is a transcriptional regulator whose overexpression is associated with metastatic potential and malignant proliferation^58^. MICAL2 is involved in modifying the cellular cytoskeleton, and in BC, its overexpression is associated with cell migration via the EGFR signaling pathway^59^. While the expression of *BHLHE40* in luminal tumors has been noted due to hypomethylation^57^ and the expression was reported to increase between normal and invasive tissues^60^, the relationship between BHLHE40 and downstream targets has not been extensively explored between subtypes and within the LM population. More notably the relationship between the TF BHLHE40 and the downstream gene *MICAL2* has not been reported in BC. As noted here, the integration of chromatin accessibility and gene regulation can distinguish the relationships between the progenitor populations and different subtypes of tumor cells and highlights specific TF regulatory networks that define this relationship.

### Lineage-specific changes from progenitor to tumor cells

**Fig. 7:**
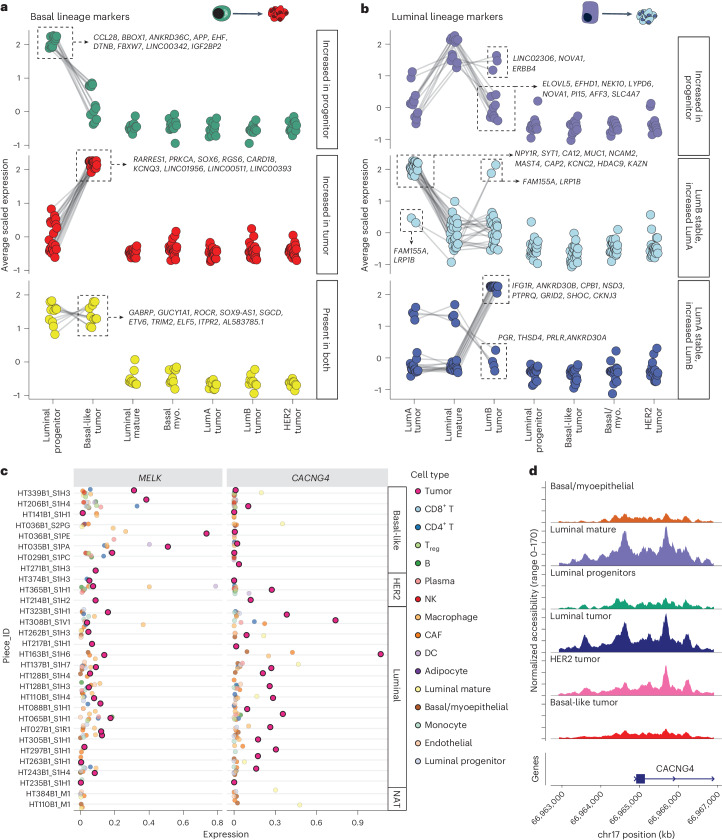
Differential markers of basal-like and luminal BC lineage. **a**, Dot-plots showing average scaled expression of basal-like BC lineage markers. Markers are divided into genes expressed highly in LP cells but not in basal-like BC (top), genes with increased expression in basal-like BC compared to LP cells (middle) and genes high in both groups (bottom). Gene lists are shown for specific groups. **b**, Dot-plots showing average scaled expression of luminal A/B BC lineage markers. Markers are divided into genes expressed highly in LM cells but not in luminal BC (top), genes with increased expression in luminal A BC compared to LM cells (middle) and genes with increased expression in luminal B BC compared to LM cells (bottom). Dot size indicates average scaled gene expression. Gene lists are shown for specific groups. **c**, Gene expression across cell types of cell surface tumor-specific markers: *MELK* identified for basal samples and *CACNG4* identified for luminal samples. **d**, Coverage plot of normalized snATAC-seq chromatin accessibility across the promoter region of *CACNG4* for tumor subtypes and benign breast cell types.

**Extended Data Fig. 7:**
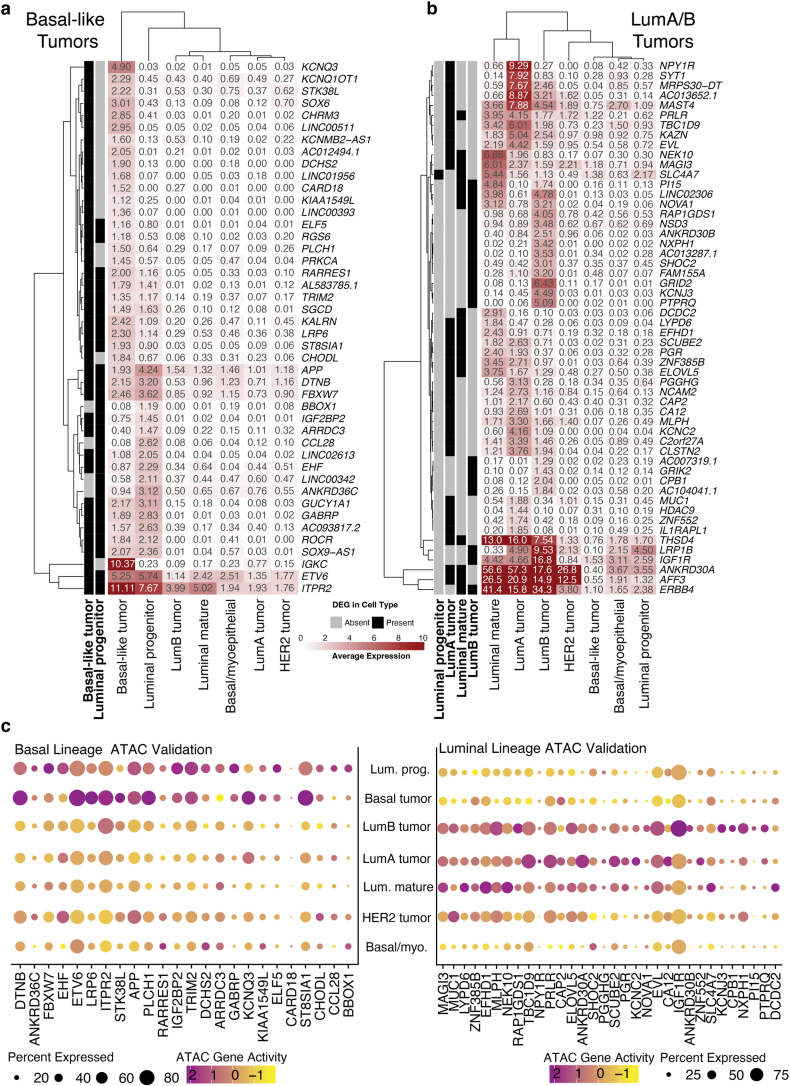
Differential gene expression (DEG) analysis by epithelial lineage. **a**) Average expression of differentially expressed genes specific to the basal tumor and/or luminal progenitor cell types. Columns labeled Basal_tumor and Luminal_progenitor indicate whether the gene was identified as a DEG for the respective cell type listed. Heatmap is colored and labeled by average expression of each epithelial cell type for comparison. **b**) Average expression of differentially expressed genes specific to the Luminal cell types, including: Luminal A tumor, Luminal B tumor, Luminal Mature or Luminal Progenitor. Columns labeled Luminal_progenitor, LumA_tumor, Luminal_mature, LumB_tumor indicate whether the gene was identified as a DEG for the respective cell type listed. Heatmap is colored and labeled by average expression of each epithelial cell type for comparison. **c**) Dot plot of signac ATAC gene activity values of basal (left) and luminal (right) lineage markers discovered by expression in snRNA-seq data. Data is colored by activity value and size of dot is associated with percent of cells with the associated average gene activity score.

To explore lineage-specific transcriptional changes between the putative cell of origin and tumor subtypes, we evaluated overlapping and unique differential gene expression profiles for each epithelial cell subset. Using a filtering strategy by comparing differentially expressed genes (DEGs) between related lineages (LP cells and basal tumor, LM and luminal tumors) and removing genes specific to uninvolved subsets (ex. basal myoepithelial and HER2 tumor), we identified 44 genes specific to the basal lineage and 54 genes specific to the luminal lineage (Methods) (Fig. 7a,b and Extended Data Fig. 7a,b). Expression of *CCL28*, *APP*, *EHF* and *LINC00342*, among others, is increased in LP cells relative to the basal tumor. Of note, ETS homologous factor is reported to be an anti-EMT factor^61^ and its decreased expression observed in tumor cells supports the finding that basal tumors tend to have increased EMT (epithelial-mesenchymal transition) properties relative to luminal tumors. Basal tumors on the other hand have increased expression of *PRKCA*, *SOX6*, *RGS6*, *CARD18* and several long noncoding RNAs, compared to the progenitor. The role of SOX family members, including *SOX6*, is well documented in basal-like BC^26^. The serine-threonine kinase *PRKCA* has been observed to be upregulated in BC, inversely correlated with ER expression and is a critical member of signaling networks in cancer stem cells, and thus is being explored as a therapeutic target in TNBC^62^. Finally, several genes share maintained expression between the progenitor and tumors, including *SOX9-AS1*, *GABRP* and *ELF5*. *GABRP* has been observed as an upregulated gene in TNBC and was found to maintain EGFR signaling in BC cell culture and contribute to chemoresistance in BC xenograft models^63^. ELF5 is a TF involved in mammary stem cell fate^64^. Notably, for the luminal subtype, we find that many DEGs are shared between the comparisons of luminal A subtype and LM and between luminal B subtype and LM, but not much overlap between all three groups. This suggests that different mature ductal progenitors may give rise to each luminal subtype. Regardless of luminal subtype (A/B), LM cells had increased expression of *ELOVL5*, *EFHD1*, *NEK10*, *LYPD6* and *NOVA1*, among others, relative to the tumor cells. *ERBB4*, *NOVA1* and *LINC02306* had relatively stable expression in luminal B and LM cells. Luminal A and luminal B tumors shared increased expression of *FAM155A* and *LRP1B* compared to LM cells, although to a greater extent in luminal B. Within the luminal A subtype, nuclear *LRP1B* was found to correlate with poor prognosis, though the mechanism of its role in carcinogenesis is unclear^65^. Luminal A and LM cells share expression of *PGR*, *THSD4*, *PRLR* and *ANKRD30A*, which are dramatically decreased in luminal B tumors. For a subset of genes with ATAC gene activity measured by Signac, we were able to validate the increased or decreased accessibility of the luminal and basal lineage genes in the ATAC data (Extended Data Fig. 7c).

**Extended Data Fig. 8:**
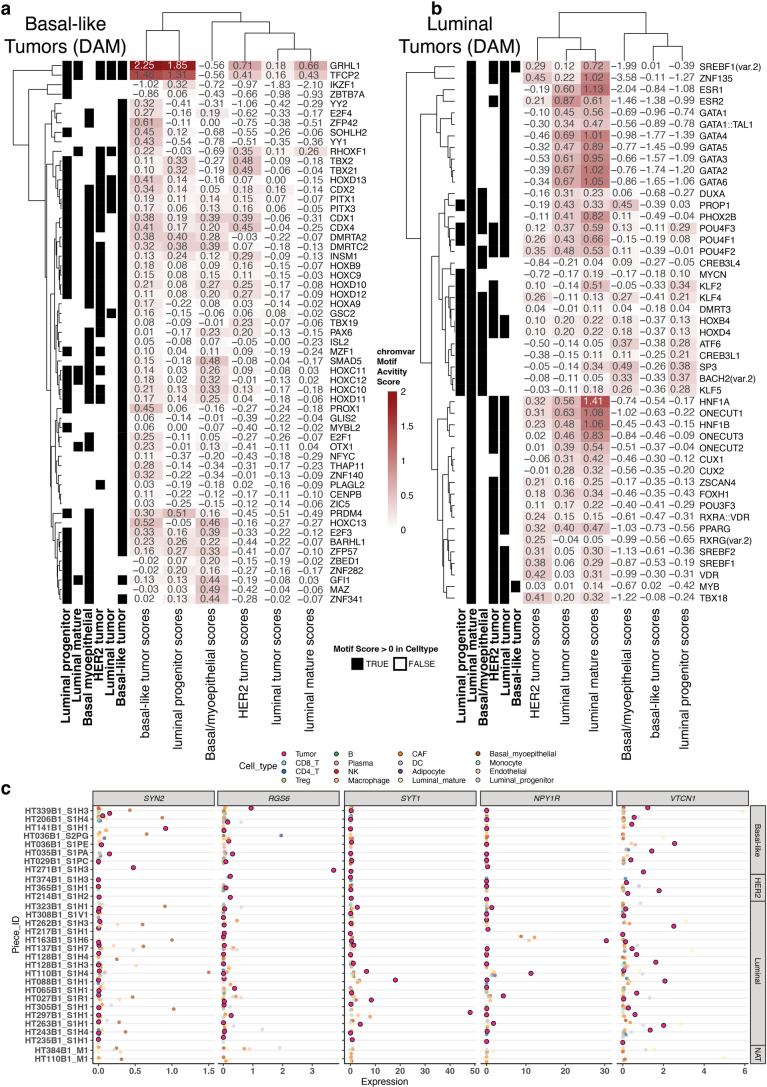
Differentially Accessible Motifs (DAM) analysis by epithelial lineage and cell-surface tumor-specific markers. **a**) Average chromvar motif activity score enriched in the basal tumor and/or luminal progenitor cell types. Columns labeled luminal progenitor (LP), luminal mature (LM), basal myoepithelial (myo), Her_tumor, Lum_tumor and Basal_tumor indicate whether the gene was identified as having a motif score greater than 0 for the respective cell type listed. Heatmap is colored and labeled by the motif activity score of each epithelial cell type for comparison. **b**) Average chromvar motif activity score enriched in the luminal tumors and/or luminal mature cell types. Columns labeled luminal progenitor (LP), luminal mature (LM), basal myoepithelial (myo), Her_tumor, Lum_tumor and Basal_tumor indicate whether the gene was identified as having a motif score greater than 0 for the respective cell type listed. Heatmap is colored and labeled by the motif activity score of each epithelial cell type for comparison. **c**) For each gene identified as a tumor-specific marker (SYN2, RGS6, SYT1, NPY1R and VTCN1) we have indicated the average expression of the gene listed in each cell type population showing an enrichment in the tumor and progenitor populations relative to other cell types.

We utilized a similar approach to evaluate differentially accessible motifs to uncover TFs that are important in maintaining lineage identity (those highly active in related lineages: LP cells and basal tumors, LM and luminal tumors) and TFs that change between benign populations and their related cancer type. We identified 57 TFs enriched in the basal lineage and 47 TFs enriched in the luminal lineage (Extended Data Fig. 8a,b). The motifs for TFs GRHL1, GRHL2, TFCP2 and HOXD13 were over-represented in open chromatin regions in both basal BC and LP cells, whereas several TFs, including YY1 and YY2, E2F1 and E2F4, SOHLH2, PROX1, OTX1, NFYC, THAP11, ZNF140 and CENPF showed further increase in enrichment in basal BC beyond that seen in LP cells. YY1 has been implicated as promoting TNBC via a long noncoding RNA mechanism leading to degradation of PTEN^66^. The role of PROX1 in BC is not well described, but it belongs to a family of genes that drive cell invasion and PROX1 has been hypothesized to drive invasiveness in colorectal cancer and Kaposi sarcoma^67,68^. Predictably, TFs related to proliferation (including E2F1, E2F4 and CENPF) were increased in basal BC compared to benign LP cells. Luminal BC and LM cells showed enrichment of ESR1 and ESR2, GATA family TFs, POU domain TFs, CUX1, CUX2 and PPARG, among others, compared to other benign breast cell types and other BC subtypes. There was overall less divergence between luminal BC and LM cells in terms of TF activity, with nearly all enriched luminal BC motifs also showing enrichment in LM cells.

### Basal-like and luminal tumor cell surface markers

**Fig. 8:**
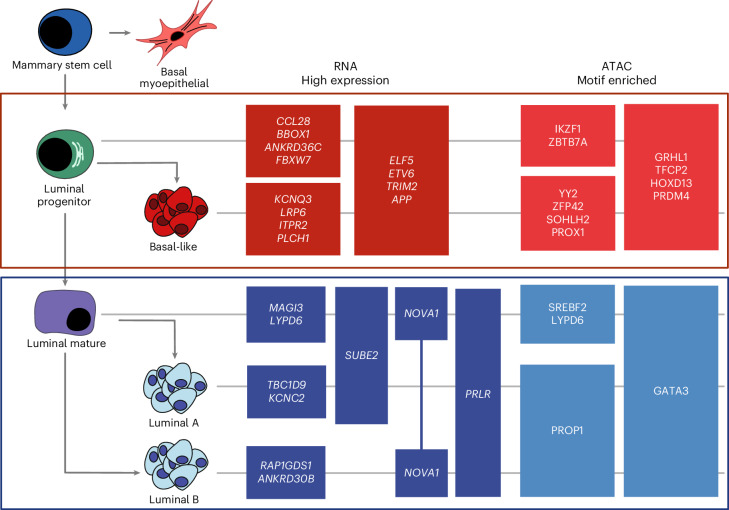
Proposed model of BC subtype progression. Model of proposed cell of origin for subtypes of BC, with key lineage-specific TF motifs and lineage defining expression markers annotated. Cell schematics were created with BioRender.com.

**Extended Data Fig. 9:**
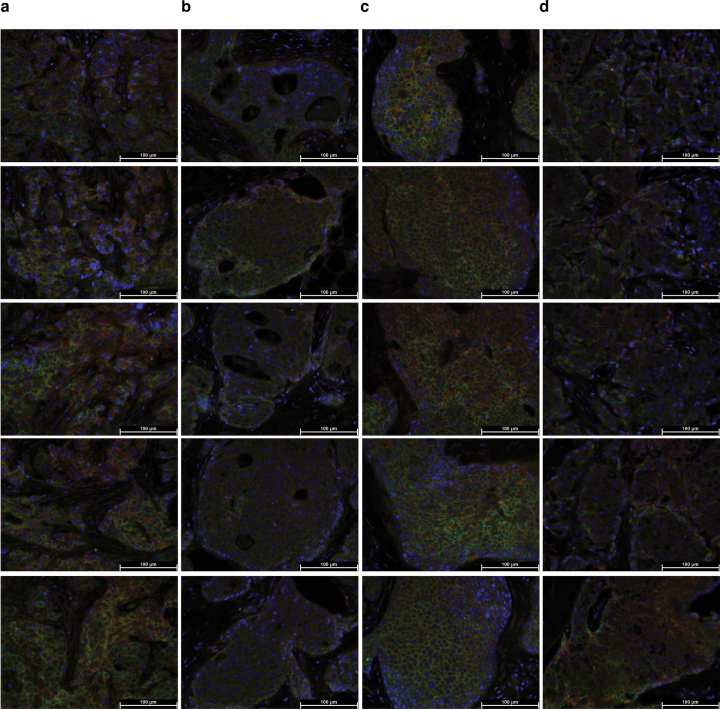
Immunofluorescence images of MELK. **a**) Immunofluorescence (IF) images of 5 representative regions of HT171B1, **b**) HT243B1, **c**) HT271B1, and **d**) HT308B1. One replicate of each indicated in figures. For all images green channel is e-cadherin, blue is DAPI and red is MELK.

**Extended Data Fig. 10:**
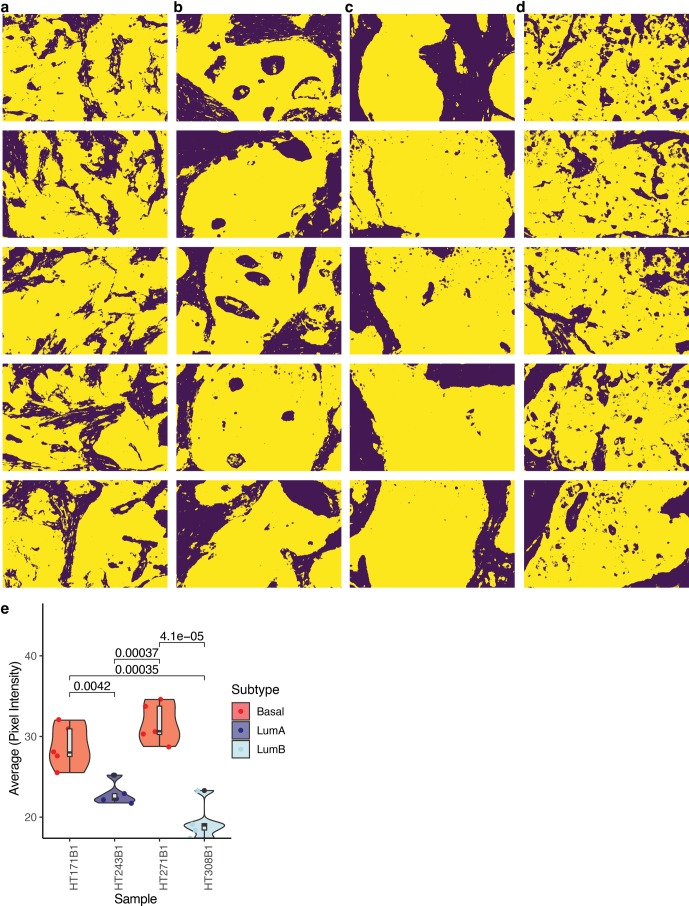
Masks associated with Immunofluorescence images of MELK. **a**) Masks of the immunofluorescence (IF) images of 5 representative regions of HT171B1, **b**) HT243B1, **c**) HT271B1, and **d**) HT308B1. One replicate of each is indicated in figures. Masks were generated based on the e-cadherin staining using adaptive thresholding. **e**) Violin plot of the average pixel intensity of each representative image from the 4 samples. P value indicated comparing the basal samples to luminal samples are derived from stat_compare_means using the method = t.test (two-sided). The boxplots show the median with 1.5 × interquartile range whiskers. For each sample, intensity from 5 regions each are indicated in the box-plot. Source data

To identify potential therapeutic targets, we searched for cell surface tumor-specific markers in samples of basal and luminal subtypes (Methods). By this analysis, we initially identified three cell surface genes expressed in basal-like samples and four in luminal A/B samples. Among those, two cell surface markers were exclusive to either basal or luminal A/B tumors: MELK in basal-like samples and CACNG4 in luminal A/B samples. MELK is a cell cycle regulator and it is known to be specifically upregulated in BC samples of basal subtype^69^ (Fig. 7c and Extended Data Fig. 8c). CACNG4 is a calcium channel subunit that was previously reported to be associated with metastasis in BC and it was also found to be highly expressed in ER^+^ BC cell lines^70^. Furthermore, we examined the promoter accessibility of those markers and we observed that the promoter region of CACNG4 is more open in luminal tumor and LM cells (Fig. 7d). The increased expression of MELK in basal samples was validated with immunofluorescence staining across four samples (two basal and two luminal) confirming the increased expression in regions of basal samples relative to luminal (Extended Data Figs. 9 and 10). VTCN1 was also identified as a tumor-enriched cell surface marker and is clinically relevant as antibody–drug conjugates targeting VTCN1 are currently being evaluated in clinical trials; however, this marker was not subtype specific. Taken together, we have started to clarify the gene regulatory networks driving the progression of a progenitor cell to luminal and basal-like BC tumor subtypes over the course of tumorigenesis with a focus on expression alterations, motif enrichment and chromatin accessibility (Fig. 8a).

## Discussion

Integration of single-cell technologies allows for high-resolution interrogation of tumor subpopulations and stromal and immune components of the tumor microenvironment. Pairing this deep-cell-level resolution with multiplex immunofluorescence imaging to provide spatial context, we identified and clarified cell precursors and transitional states and how these transitional populations are associated with chromatin accessibility. snATAC-seq reveals the transcriptional elements underlying these changes. CODEX immunofluorescence and ST provide support for these findings and tie single-nucleus findings to discrete histologic structures. Collectively, this study presents multi-omic evidence of the transcriptional programs connecting BC subtypes to distinct cells of origin. Identification of the fine changes associated with transient transitional states is not captured by bulk methods and may have implications for current treatment paradigms in BC.

BC subtyping is generally based on bulk gene expression, which is limited by prevalence of non-tumor cell types. In this work, we apply the PAM50 subtyping algorithm in tandem to bulk-RNA-seq and snRNA-seq to reliably classify even low-purity tumor samples. Chromatin accessibility from snATAC-seq also clearly separated tumors by subtype and had good concordance with bulk and snRNA-seq-based classifications and highlights transcriptional networks that underlie their gene expression profiles. In addition to known TFs such as GATA3 and FOXP1, we identify HNF1A as a TF specific to luminal A/B breast tumors and LM breast duct cells. HNF1A is not well studied in BC, but is important in colon and pancreatic cancer development and drives PI3K/AKT signaling in esophageal cancer^71–73^. An antisense product of *HNF1A*, *HNF1A-AS1*, is upregulated in BC and increases proliferation, migration, invasion and tamoxifen sensitivity in multiple BC cell lines^74^. While this work primarily categorizes BC by PAM50 subtype, there is a lower rate of PR positivity in luminal B compared to luminal A tumors in our data, which could confound findings specific to only one of the luminal subtypes. The lower rate of PR positivity in luminal B tumors is well established^75^ and further validation could be undertaken in PR^+^ cohorts. In basal-like BC, we show increased chromatin accessibility for the motifs of known TFs, including SOX4, SOX9 and E2F family proliferation-related TFs. Additionally, we highlight TEAD family TF motifs as highly enriched in the open chromatin of basal-like BC and GRHL1/2 and TFCP2 as enriched in both basal-like tumors and LP cells. TEAD TFs are associated with YAP/TAZ transcriptional activators to drive expression of Hippo pathway genes in BC^76^. GRHL2, a member of the Grainyhead TF family, is involved in maintenance of the epithelial phenotype and has been considered a tumor suppressor^77^; however, our results suggest that GRHL2 activity is maintained in basal-like tumors. These results are consistent with the proposed oncogenic role of GRHL2; loss of GRHL2 in the BC cell line MCF7 is associated with decreased proliferation and GRHL2 can also suppress the death receptors FAS and DR5 (refs. ^78,79^). TFCP2 has known oncogenic roles in hepatocellular carcinoma, pancreatic adenocarcinoma and BC; it has roles in maintaining cell stemness and in EMT and angiogenesis^80^.

Epigenetic features such as DNA methylation and histone modification have been used to track cell lineage and identify cells of origin^27–30^. Because ATAC-seq shows the footprints of TF programs, it complements gene expression data in discerning tumor pathogenesis and carries implications for therapeutic targets to modulate these programs. Chromatin accessibility as assessed by snATAC-seq has key advantages beyond gene expression alone; the maintenance of accessibility patterns across cell types allows for clearer delineation of cell lineage, and it points to upstream effectors of gene expression changes, which may themselves be therapeutic targets. In an effort to clarify cells of origin of BC, experimental data robustly support the most widely accepted model that luminal BC arises from a mature ER^+^ breast duct cell, whereas basal-like BC arises from an LP cell in the same lineage^8,43^. This study adds to our understanding by incorporating many patients with diverse tumor types and mutational spectra and supports the established model that basal-like breast tumors arise from LP cells, whereas luminal A/B tumors arise from LM cells. The addition of snATAC-seq in many samples adds orthogonal evidence for this conclusion and has not previously been reported. Combining snRNA-seq and snATAC-seq, we implicate several transcriptional programs which are maintained in breast tumors and their proposed cell of origin and have not been extensively reported previously. In both LM duct cells and luminal tumors, BHLHE40 was predicted to regulate coexpressed genes. The precise role of BHLHE40 in BC has not been well described; its role in the luminal lineage may shed light on a targetable pathway in BC treatment or prevention. Further, by evaluating differentially accessible motifs and expression features of each epithelial cell type, we were able to distinguish expression signatures that are altered over the course of tumorigenesis. While we were able to rigorously define the changes between progenitors and tumor cells in luminal A/B and basal-like tumors, we were not able to do so for HER2-enriched tumors. At present we are currently underpowered to address this question likely due to the low sampling size of HER2-enriched tumors in our cohort (three patients with single-nucleus data). Limited analyses of these few samples show shared transcriptional features with luminal BC. Future studies focusing on HER2-enriched samples can utilize a similar framework to evaluate the proposed cell of origin for this unique subtype. Additionally, our data include samples from patients receiving a wide variety of systemic therapies as well as treatment-naive samples; additional studies could explore how transcriptional programs in BC are impacted by treatment.

It is well known that substantial immune infiltration is seen in a subset of breast tumors and that the likelihood of this phenomenon varies by subtype^81,82^. In particular, some basal-like breast tumors are observed to harbor a dense immune infiltrate and this finding is a positive prognostic feature when it is found^82,83^. Recently, this enrichment in immune infiltration in basal-like BC has led to the approval of pembrolizumab (anti-PD-1 immunotherapy) plus chemotherapy for early-stage TNBC^84^. Using scRNA-seq, we can finely dissect the immune landscape within different breast tumors. We observe significantly more CD8^+^ exhausted T cells in basal-like tumors compared to other subtypes, which has been observed previously^85,86^. Ligand–receptor interaction predicts that CTLA4 expressed on CD8^+^ T cells directly interacts with CD80 or CD86 expressed on multiple myeloid cell types. CCL3 on CD8^+^ T cells was also predicted to interact with CCR1 or CCR5 on myeloid cells and CXCL13 on CD8^+^ T cells was predicted to interact with CXCR5 on B cells or ACKR4 on cancer-associated fibroblasts. All three of the genes, *CTLA4*, *CCL3* and *CXCL13*, are more highly expressed on CD8^+^ T cells in basal-like tumors compared to those of other subtypes. CTLA4 provides a negative modulatory signal to T cells interacting with its primary ligands, CD80 or CD86, on an antigen-presenting cell, and is well described as a key component of tumor immune evasion^87,88^. Anti-CTLA4 blockade with ipilimumab or tremelimumab has been proven effective as immunotherapy in a diverse group of non-BC tumor types, but is not used in standard therapy for BC. The success of the KEYNOTE-522 trial of neoadjuvant pembrolizumab, an anti-PD-1 monoclonal antibody, in TNBC demonstrates a role for immune-checkpoint blockade in BC^84^. Our work suggests that anti-CTLA4 therapies may also be effective in modulating the antitumor immune response, particularly in basal-like BC.

Spatial profiling of cellular proteins with CODEX complements our understanding gleaned from single cell or single-nucleus-based approaches. In this work, we show that GATA3 and c-KIT are markers of LM and LP cells, which are maintained in the transition from benign precursor to invasive BC. Future work will be focused on prospective validation of markers for tumor and benign populations as this may reveal new potential drug targets specific to key populations along the BC evolutionary lineage.

## Methods

### Human specimens and clinical data

All samples were collected with informed consent in concordance with institutional review board (IRB) approval. Primary breast carcinoma samples were collected during surgical resection and verified by standard pathology (IRB protocol 201108117). Blood was collected at the time of surgery into vacuum tubes containing ethylenediaminetetraacetic acid (EDTA) (BD Bioscience). Cells were isolated by Ficoll-density centrifugation and frozen in fetal bovine serum with 5% dimethyl sulfoxide. Clinical data were captured in accordance with IRB protocol 20108117 at the time of informed consent and entered into the REDCap database.

### Statistics and reproducibility

Relevant statistics are referred to in each of the associated methods sections. We did not use statistical methods to predetermine a sample size and patients were not randomly selected as patients were enrolled as they entered the clinic. We excluded samples that did not pass sample preparation QC. Further information on research design is available in the Nature Research Reporting Summary linked to this article. Data distribution was assumed to be normal but this was not formally tested. Data collection and analysis were not performed blind to the conditions of the experiments.

### Human sample processing

After verification by an attending pathologist, a 1.5 × 1.5 × 0.5-cm portion of the tumor was removed, photographed, weighed and measured. Each piece was then subdivided into 6–9 pieces (depending on the original size) and then further subdivided into four transverse cut pieces. Pieces were then placed into formalin, snap frozen in liquid nitrogen, DMEM and formalin, respectively. Relevant protocols can be found at https://www.protocols.io/view/biospecimen-collection-and-processing-2-0-bp2l6b3bzgqe/v1(ref. ^89^). As per the institutional requirement, pathology restricts the sampling of any tumors to 2 cm or above with no restriction on maximal tumor size or burden.

### Pathologic parameters and assessment

Each tumor that was subdivided into smaller increments was subjected to H&E stain and was assessed by a pathologist for the following parameters: tumor differentiation and grade, percentage of tumor-infiltrating lymphocytes, lymphovascular invasion and perineural invasion. Tumor viability was also assessed by the presence or absence of necrosis. Both slices of each tumor piece, both L1 and L4 when available were subjected to assessment.

### Mouse sample collection and processing

B6.FVB-Tg(MMTV-PyVT) 634Mul/LellJ (strain 022974) female mice were purchased from the Jackson Laboratory. Mice were killed by carbon dioxide asphyxiation and five pairs of mouse mammary glands were collected at 12 weeks old. For each pair, the left mammary gland was flash frozen in liquid nitrogen and the right glands were fixed in 10% neutral buffered formalin (Epredia, 5725) then embedded in paraffin. The five left mammary glands were pooled together for snRNA-seq and snATAC-seq sample preparation. The paraffin-embedded glands were used for H&E staining. All animal experiments were approved by the Washington University in Saint Louis Institutional Animal Care and Use Committee office.

### Genomic DNA and RNA extraction

Tumor tissues and corresponding normal tissues were obtained from surgically resected specimens and after a piece was removed for fresh single-cell preparation the remaining sample was snap frozen in liquid nitrogen and stored at −80 °C. Before bulk-RNA/DNA extraction, samples were cryopulverized (Covaris) and aliquoted for bulk extraction methods. Genomic DNA was extracted from tissue samples with either the DNeasy Blood and Tissue kit (QIAGEN, 69504) or the QIAamp DNA Mini kit (QIAGEN, 51304). Total RNA was extracted with TRI reagent (Millipore Sigma, T9424) and treated with DNase I (QIAGEN, 79254) using an RNeasy MinElute Cleanup kit (QIAGEN, 74204). RNA integrity was evaluated using either a Bioanalyzer (Agilent Technologies) or TapeStation (Agilent Technologies). Germline genomic DNA was purified from cryopreserved peripheral blood mononuclear cells using the QIAamp DNA Mini kit (QIAGEN, 51304) according to the manufacturer’s instructions. DNA quantity was assessed by fluorometry using the Qubit dsDNA HS Assay (Thermo Fisher Scientific, Q32854) according to the manufacturer’s instructions (Thermo Fisher Scientific). Relevant protocols can be found at https://www.protocols.io/view/bulk-dna-extraction-ding-lab-bsnhndb6, https://www.protocols.io/view/bulk-rna-isolation-ding-bsnfndbn (refs. ^90,91^).

### Whole-exome sequencing

A total of 100–250 ng of genomic DNA was fragmented on the Covaris LE220 instrument targeting 250-bp inserts. Automated dual indexed libraries were constructed with the KAPA Hyper library prep kit (Roche) on the SciClone NGS platform (PerkinElmer). Up to ten libraries were pooled at an equimolar ratio by mass before hybrid capture targeting a 5-µg library pool. The library pools were hybridized with the xGen Exome Research Panel v.1.0 reagent (IDT Technologies) that spans a 39-Mb target region (19,396 genes) of the human genome. The libraries were hybridized for 16–18 h at 65 °C followed by a stringent wash to remove spuriously hybridized library fragments. Enriched library fragments were eluted and PCR cycle optimization was performed to prevent over amplification. The enriched libraries were amplified with KAPA HiFi master mix (Roche) before sequencing. The concentration of each captured library pool was accurately determined through qPCR utilizing the KAPA library Quantification kit according to the manufacturer’s protocol (Roche) to produce cluster counts appropriate for the Illumina NovaSeq-6000 instrument. Then, 2 × 150 paired-end reads were generated, targeting 12 Gb of sequence to achieve ~100× coverage per library.

### RNA sequencing

Total RNA integrity was determined using Agilent Bioanalyzer or 4200 TapeStation. Library preparation was performed with 500 ng to 1 μg total RNA. Ribosomal RNA was blocked using FastSelect reagents (QIAGEN) during cDNA synthesis. RNA was fragmented in reverse transcriptase buffer with FastSelect reagent and heating to 94 °C for 5 min, 75 °C for 2 min, 70 °C for 2 min, 65 °C for 2 min, 60 °C for 2 min, 55 °C for 2 min, 37 °C for 5 min and 25 °C for 5 min. mRNA was reverse transcribed to yield cDNA using SuperScript III RT enzyme (Life Technologies, per manufacturer’s instructions) and random hexamers. A second strand reaction was performed to yield ds-cDNA. cDNA was blunt ended, had an A base added to the 3′ ends and then had Illumina sequencing adaptors ligated to the ends. Ligated fragments were then amplified for 15 cycles using primers incorporating unique dual index tags. Fragments were sequenced on an Illumina NovaSeq-6000 S4 instrument generating approximately 30 million paired-end 2 × 150 reads per library.

### Single-cell suspension preparation

For each tumor, approximately 15–100 mg of 2–4 sections of each tumor and/or normal piece of tissue were cut into small pieces using a blade and processed separately. Enzymes and reagents from the human tumor dissociation kit (Miltenyi Biotec, 130-095-929) were added to the tumor tissue along with 1.75 ml DMEM. The resulting suspension was loaded into a gentleMACS C-tube (Miltenyi Biotec, 130-093-237) and subject to the gentleMACS Octo Dissociator with Heaters (Miltenyi Biotec, 130-096-427). After 30–60 min on the heated dissociation program (37h_TDK_1), samples were removed from the dissociator and filtered through a 40-μM Mini-Strainer (PluriSelect, 43-10040-60) or 40-μm nylon mesh (Fisher Scientific, 22-363-547) into a 15-ml conical tube on ice. The sample was then spun down at 400*g* for 5 min at 4 °C. After removing supernatant, when a red pellet was visible the cell pellet was resuspended using 200 μl to 3 ml ACK Lysis Solution (Thermo Fisher, A1049201) for 1–5 min. To quench the reaction, 10 ml PBS (Corning, 21-040-CM) with 0.5% BSA (Miltenyi Biotec, 130-091-376) was added and spun down at 400*g* for 5 min at 4 °C. After removing supernatant, cells were resuspended in 1 ml PBS (Corning, 21-040-CM) with 0.5% BSA. Live and dead cells were visualized using Trypan blue. If greater than 40% of dead cells were present, the sample was spun down at 400*g* for 5 min at 4 °C and subjected to the dead cell removal kit (Miltenyi Biotec, 130-090-101). Finally, the sample was spun down at 400*g* for 5 min at 4 °C and resuspended in 500 μl to 1 ml PBS with 0.5% BSA to a final concentration of 700 to 1,500 cells per μl. A step-by-step protocol is found at https://www.protocols.io/view/wu-sc-prep-protocol-for-solid-tumors-v2-1-yxmvmkp5bg3p/v1 (ref. ^92^).

### Single-cell library prep and sequencing

Utilizing the Chromium Next GEM Single Cell 3′ GEM, Library & Gel Bead kit v.3.3 and Chromium instrument, approximately 17,500 to 25,000 cells were partitioned into nanoliter droplets to achieve single-cell resolution for a maximum of 10,000–15,000 individual cells per sample (10x Genomics). The resulting cDNA was tagged with a common 16-nt cell barcode and 10-nt unique molecular identifier (UMI) during the reverse transcriptase (RT) reaction. Full-length cDNA from poly-A mRNA transcripts was enzymatically fragmented and size selected to optimize the cDNA amplicon size (approximately 400 bp) for library construction (10x Genomics). The concentration of the 10x single-cell library was accurately determined through qPCR (Kapa Biosystems) to produce cluster counts appropriate for the HiSeq 4000 or NovaSeq-6000 platform (Illumina). The 26 × 98-bp sequence data were generated targeting 50,000 read pairs per cell, which provided digital gene expression profiles for each individual cell.

### Single-nuclei RNA and ATAC library preparation and sequencing

Approximately 20–30 mg of cryopulverized powder from BRCA specimens was resuspended in 2 ml lysis buffer (10 mM Tris-HCl (Thermo Fisher, 15567027) (pH 7.4); 10 mM NaCl (Themo Fisher, AM9759); 3 mM MgCl_2_ (Thermo Fisher, AM9530G) and 0.1% NP-40 (Sigma, 74385-1L)) plus 0.1 U μl^−1^ RNase Inhibitor (Invitrogen, AM2696). This suspension was pipetted gently 6–8 times, incubated on ice for 30 s and pipetted again 4–6 times. The lysate containing free nuclei was filtered through a 40-μm cell strainer. We washed the filter with 1 ml Wash and Resuspension buffer (1× PBS (Corning, 21-040-CM) + 2% BSA (Miltenyi Biotec, 130-091-376) + 0.2 U μl^−1^ RNase inhibitor) plus 0.1 U μl^−1^ RNase Inhibitor and combined the flow through with the original filtrate. After a 6-min centrifugation at 500*g* and 4 °C, the nuclei pellet was resuspended in 300 μl Wash and Resuspension buffer plus 0.1 U μl^−1^ RNase Inhibitor. After staining with 1 μl 7AAD (ATAC or multiome) or DRAQ5 (RNA) the nuclei were further purified by FACS. FACS-purified nuclei were centrifuged again and resuspended in a small volume (~30 μl Wash and Resuspension buffer plus 0.1 U μl^−1^ RNase Inhibitor). After counting and microscopic inspection of nuclei quality, the nuclei preparation was diluted to ~1,000 nuclei per μl. Then, ~20,000 nuclei were used for snRNA-seq by the 10x Chromium platform. For snRNA, we loaded the single nuclei onto a Chromium Next GEM Chip G kit and processed them through the Chromium Controller to generate GEMs (gel beads in emulsion). For a subset of samples with joint snRNA and snATAC the multiome kit, Chromium Next GEM Single Cell Multiome ATAC + Gene Expression was used. For ATAC-only samples FACS-purified nuclei were centrifuged again and resuspended in 5 μl Diluted Nuclei Buffer. After counting and microscopic inspection of nuclei quality, the nuclei preparation, ~10,000 nuclei were used for snATAC-seq by the 10x Chromium platform. We loaded the single nuclei onto a Chromium Next GEM Chip H kit and processed them through the Chromium Controller to generate GEMs. After that, post-GEM-RT Cleanup was performed with target cell recovery ≥2,000. We then prepared the sequencing libraries following the manufacturer’s protocol. All sequencing was performed on an Illumina NovaSeq-6000 S4 flow cell. The libraries were pooled and sequenced using the XP workflow according to the manufacturer’s protocol with a 28 × 8 × 98-bp sequencing recipe. The resulting sequencing files were available in FASTQ format per sample after demultiplexing. A step-by-step protocol is found at https://www.protocols.io/view/wu-sn-prep-protocol-for-solid-tumors-snrna-protoco-14egn7w6zv5d/v1 (ref. ^93^).

### Fluorescence-activated cell sorting

Depending on the pellet size, 100–500 μl nuclei suspension in the wash buffer (2% BSA + 1× PBS + RNase inhibitor) was stained with DRAQ5 or 7AAD for RNA or ATAC sequencing, respectively (7AAD was used for multiome processing). Namely, snRNA-seq nuclei were stained with 1 μl DRAQ5 per 300 μl of the sample and snATAC-seq nuclei were stained with 1 μl 7AAD per 500 μl sample. Sorting gates were based on size, granularity and dye staining signal.

### Immunofluorescence and microscopy

Fresh tissues were fixed in 10% neutral buffered formalin (Epredia, 5725) at room temperature overnight but for less than 24 h. Tissues were then dehydrated, infiltrated with wax and embedded into paraffin blocks. After tissues were processed into formalin-fixed paraffin-embedded blocks, 5-μm sections were cut and placed on glass slides. Next, sections were deparaffinized and rehydrated, followed by antigen retrieval using Tris EDTA buffer, pH 9 (Genemed, 10-0046) or 1× sodium citrate, pH 6 (Sigma, C9999) according to the manufacturer’s recommendation for specific antibodies. Then, sections were blocked with 100 mM glycine for 20 min, followed by blocking with 10% normal serum and 1% BSA for 1 h at room temperature. A negative control and a secondary antibody control were used in each experimental setting. Primary antibodies for MELK (Thermo Fisher, MA517120) and E-cadherin (R&D, AF748) were applied on sections at 4 °C overnight, followed by the incubation of appropriate secondary antibodies the next day. Images were collected using a Leica DMi8 microscope.

### CODEX preparation and imaging

Carrier-free monoclonal or polyclonal anti-human antibodies were purchased from different companies (Supplementary Table 6) and verified using immunofluorescence (IF) staining in multiple channels. After screening, antibodies were conjugated using an Akoya Antibody Conjugation kit (Akoya Biosciences, SKU 7000009) with a barcode (Akoya Biosciences) assigned based on the IF staining results. Several common markers were directly purchased through Akoya Biosciences (Supplementary Table 6). CODEX staining and imaging were performed according to the manufacturer’s instruction (CODEX User Manual, Rev C). In brief, 5-μm formalin-fixed paraffin-embedded sections were placed on APTES (Sigma, 440140)-coated coverslips and baked at 60 °C overnight before deparaffinization. The next day, tissues were incubated in xylene, rehydrated in ethanol and washed in ddH_2_O before antigen retrieval with TE buffer, pH 9 (Genemed, 10-0046) in boiling water for 10 min in a rice cooker. The tissues were then blocked using blocking buffer (CODEX staining kit, SKU 7000008) and stained with the marker antibody panel (Supplementary Table 6) to a volume of 200 µl for 3 h at room temperature in a humiliated chamber. Imaging of the CODEX multicycle experiment was performed using a Keyence fluorescence microscope (model BZ-X810) equipped with a Nikon CFI Plan Apo λ ×20/0.75 objective, the CODEX instrument (Akoya Biosciences) and CODEX Instrument Manager (Akoya Biosciences). Exposure times, dilutions and the order of markers per cycle are listed in Supplementary Table 6. The raw images were then stitched and processed using the CODEX processor (Akoya Biosciences). After multiplex imaging was completed, H&E staining was performed on the same tissue.

### ST preparation and sequencing

OCT-embedded tissues were cryosectioned and placed on Visium Spatial Gene Expression Slide following Visium Spatial Protocols-Tissue Preparation Guide (10x Genomics, CG000240 Rev A). Briefly, fresh tissues were coated carefully and thoroughly with room temperature OCT without any bubbles. OCT-coated tissues were then placed on a metal block chilled in dry ice until the OCT turned solidified and white. After an RNA quality check using TapeStation and morphology check using H&E staining for the OCT-embedded tissues, blocks were scored into proper size that fit the Capture Areas and then sectioned into 10-μm sections. After the tissue placement into the Capture Area, sections were fixed in methanol, stained with H&E and imaged at ×20 magnification using the brightfield imaging setting on Leica DMi8 microscope. Tissues were then permeabilized for 18 min and ST libraries were constructed following Visium Spatial Gene Expression Reagent Kits User Guide CG000239 Rev A (10x Genomics). Briefly, cDNA was reverse transcribed from the poly-adenylated mRNA which was captured by the primers on the slides. Next, the second strand was synthesized and denatured from the first strand. Free cDNA was then transferred from slides to tubes for further amplification and library construction. Libraries were sequenced on the S4 flow cell of Illumina NovaSeq-6000 system. A step-by-step protocol is found at 10.17504/protocols.io.x54v9d3opg3e/v1 (ref. ^94^).

### Quantification and statistical analysis

#### Genomic data analysis

##### Tumor-normal somatic variant calling

Somatic variants were called from WES tumor and normal paired BAMs using somaticwrapper v.1.6, a pipeline designed for detection of somatic variants from tumor and normal exome data. The pipeline merges and filters variant calls from four callers: Strelka v.2.9.2 (ref. ^95^), VarScan v.2.3.8 (ref. ^96^), Pindel v.0.2.5 (ref. ^97^) and MuTect v1.1.7 (ref. ^98^). Single-nucleotide variant (SNV) calls were obtained from Strelka, VarScan and MuTect. Indel calls were obtained from Stralka2, VarScan and Pindel. The following filters were applied to get variant calls of high confidence: normal VAF ≤ 0.02 and tumor VAF ≥ 0.05, read depth in tumor ≥14 and normal ≥8, indel length <100 bp, all variants must be called by two or more callers, all variants must be exonic and exclude variants in dbSNP but not in COSMIC.

##### Tumor-only somatic variant calling

Tumor-only somatic variants were called using Mutect2 (v.4.1.2.0) best-practice pipeline (gatk.broadinstitute.org) with the GDC Panel of Normal (PON) data (gdc.cancer.gov/about-data/data-harmonization-and-generation/gdc-reference-files; gatk4_mutect2_4136_pon.vcf.tar). To further reduce false positives, we kept the mutation sites with ≥20× coverage, >3 reads and ≥0.1 tumor VAF, which was supported by bam-readcount (https://github.com/genome/bam-readcount).

##### Somatic variant rescue

In some tumor cases, we called somatic variants of driver genes for some pieces, but not for all pieces. Therefore, we used bam-readcount to rescue those variants for the piece(s) without them. We kept default parameters for bam-readcount, except setting –min-mapping-quality as 20 and –min-base-quality as 20.

##### Germline variant calling and annotation

Germline variant calling was performed using an in-house pipeline GermlineWrapper v.1.1, which implements multiple tools for the detection of germline INDELs and SNVs. Germline SNVs were identified using VarScan v.2.3.8 (with parameters –min-var-freq 0.10–p-value 0.10, –min-coverage 3–strand-filter 1) operating on a mpileup stream produced by SAMtools v.1.2 (with parameters -q 1 -Q 13) and GATK v.4.0.0.0 (ref. ^99^) using its haplotype caller in single-sample mode with duplicate and unmapped reads removed and retaining calls with a minimum quality threshold of 10. Germline INDELs were identified using VarScan (version and parameters as above) and GATK (version and parameters as above) in single-sample mode. SNVs were based on the union of raw GATK and VarScan calls. We required that indels were called by Pindel or at least two out of the three callers (GATK, VarScan and Pindel). Cutoffs of minimal 10× coverage and 20% VAF were used in the final step to report high-quality germline variants. All resulting variants were limited to the coding region of the full-length transcripts obtained from Ensembl release 100 plus additional two base pairs flanking each exon to cover splice donor/acceptor sites. We also required variants to have an allelic depth ≥5 for the alternative allele in both tumor and normal samples and filtered out any indels longer than 100 bp.

##### Germline variant pathogenic classification

Germline variants called with GermlineWrapper were annotated with the Ensembl Variant Effect Predictor^100^ (v.100 with default parameters, except where –everything) and their pathogenicity was determined with our automatic pipeline CharGer^48^ (v.0.5.4 with default CharGer scores; https://github.com/ding-lab/CharGer/tree/v0.5.4), which annotates and prioritizes variants based on the American College of Medical Genetics and Genomics–Association for Molecular Pathology (ACMG–AMP) guidelines^101^. The detailed implementation, score of each evidence level and parameters used are as previously described^102^.

We selected rare variants with ≤0.05% allele frequency in gnomAD (r2.1.1) or 1000 Genomes^103^. We also performed readcount analysis using bam-readcount (https://github.com/genome/bam-readcount; v.0.8 with parameters -q 10, -b 15) in both normal and tumor samples. We required variants to have at least five counts of the alternative allele and a VAF of at least 20% in both tumor and normal. Variants affecting known cancer predisposition genes (previously described elsewhere^102^) were manually reviewed with the Integrative Genomics Viewer software (21221095; v.2.8.2). We considered variants to be ‘pathogenic’ if they were known pathogenic variants in ClinVar; ‘likely pathogenic’ if CharGer score >8; and a ‘prioritized variant of uncertain significance’ if CharGer score >4.

Variants called in cases where a normal sample was not available (tumor only) were further filtered for removal of potential somatic events. Variants in these cases called by our germline pipeline which were also called by our somatic pipeline were filtered out, as well as variants not previously reported in gnomAD or reported in COSMIC (extracted from Variant Effect Predictor annotation).

##### RNA quantification

We used an in-house bulk-RNA-seq analysis pipeline for quantification (https://github.com/ding-lab/HTAN_bulkRNA_expression). In brief, for each sample, the raw sequence reads were aligned into BAM files using STAR (v.2.7.4a) two-pass alignment with GRCh38 as the reference. The resulting BAM files were then quantified as a raw count matrix using feature counts (subread, v.2.0.1). For both alignment and quantification, gene annotations were based on Gencode v.34. The raw counts were then converted to FPKM-UQ based on GDC’s formula and then log_2_ transformed with one pseudocount (https://docs.gdc.cancer.gov/Data/Bioinformatics_Pipelines/Expression_mRNA_Pipeline/#upper-quartile-fpkm).

##### Expression-based subtyping

Bulk expression subtyping was performed according to the methods detailed by Parker et al.^104^ using the log_2_ upper quartile-normalized FPKM reads for all bulk-RNA-seq samples. Median values of all 50 PAM50 genes were provided for median adjustment. To minimize the influence of any one sample on subtype calls, median values for the 50 PAM50 genes were bootstrapped using a subset of the bulk-RNA-seq data comprising all 17 ER^−^ samples and an equal number of randomly selected ER^+^ samples. PAM50 subtype assignments were called for all samples using 1,000 such sets of median values and a final subtype assignment for each sample was the subtype most commonly called across these iterations. The PAM50 subtype-calling algorithm was run using code provided by Parker et al.^104^ using R v.4.0.2.

#### scRNA-seq and snRNA-seq quantification and analysis

##### scRNA-seq and snRNA-seq data preprocessing

For each sample, we obtained the unfiltered feature–barcode matrix per sample by passing the demultiplexed FASTQs to the CellRanger v.6.0.2 ‘count’ command using default parameters and the prebuilt GRCh38 genome reference (refdata-gex-GRCh38-2020-A; GRCh38 and Ensembl 93). Seurat v.4.1.0 (refs. ^105,106^) was used for all subsequent analysis. First, a series of quality filters were applied to the data to remove those barcodes that fell into any one of these categories recommended by Seurat: too few total transcript counts (<300); possible debris with too few genes expressed (<200) and too few UMIs (<1,000); possible more than one cell with too many genes expressed (>10,000) and too many UMIs (>10,000); possible dead cell or a sign of cellular stress and apoptosis with too high proportion of mitochondrial gene expression over the total transcript counts (>10%). Doublets were filtered out using Scrublet (https://github.com/AllonKleinLab/scrublet). Scrublet was run on each sample separately with the following parameter settings: expected_doublet_rate = 0.06, min_counts = 2, min_cells = 3, min_gene_variability_pctl = 85 and n_prin_comps = 30. The doublet score threshold was adjusted manually. We constructed a Seurat object using the unfiltered feature–barcode matrix for each sample. Each sample was scaled and normalized using Seurat’s ‘SCTransform’ function to correct for batch effects (with parameters vars.to.regress = c(‘nCount_RNA’, ‘percent.mito’) and variable.features *n* = 2,000). Any merged analysis or subsequent subsetting of cells/samples underwent the same scaling and normalization method. Cells were clustered using the original Louvain algorithm and top 30 PCA dimensions via ‘FindNeighbors’ and ‘FindClusters’ (with parameters: resolution = 0.5) functions. The resulting merged and normalized matrix was used for the subsequent analysis. Each sample was scaled and normalized using Seurat’s ‘SCTransform’ function to correct for batch effects (with parameters: vars.to.regress = c(‘nCount_RNA’, ‘percent.mito’), variable.features.n = 3,000). We then merged all samples and repeated the same scaling and normalization method. All cells in the merged Seurat object were then clustered using the original Louvain algorithm (Blondel et al. 2008) and top 30 PCA dimensions via ‘FindNeighbors’ and ‘FindClusters’ (with parameters: resolution = 0.5) functions. The resulting merged and normalized matrix was used for subsequent analysis.

##### scRNA-seq and snRNA-seq cell type annotation

The main cell types were assigned to each cluster by manually reviewing the expression of a comprehensive set of marker genes. The marker genes used were:

*CCL5, FYN, CCL4, IL7R, GNLY* (T/NK cells); *CD8B, CD8A, CD3E, CD3D* (CD8^+^ T cells); *CD4, CD3E, CD3D, SELL, CCR7, IL7R, TCF7, LEF1* (CD4^+^ T cells); *XCL2, XCL1, KLRF1, KIR2DL3, IL2RB, CD7, KLRB1, KLRD1, GZMA, PRF1, CD160, NCAM1* (NK cells); NK markers and *CD3E, CD3D* (NKT cells); *FABP4, VWF, ACKR1, LDB2, PECAM1* (endothelial cells); *COL1A2, COL1A1, COL3A1, SFRP2, DCN, SMOC2, ITGBL1, FBLN1, CDH11, PDGFRA, SVEP1, PDPN, LRRC15, CILP, LUM, MFAP5, FBLN2, OLFML3, RNASE4* (mCAF); *MGP, SCGB2A2, SLPI, LTF, PTN, KIT, ALDH1A3* (LP cells); *SPP1, APOE, LYZ, APOC1, HLA-DRA* (monocytes/macrophages); *ATP1B2, DES, AOC3, NDUFA4L2, MCAM, HIGD1B, CPE, KCNJ8, ABCC9, IGFBP7, TAGLN, ACTA2, MYL9, CALD1* (vCAF); *CFD, DCN, GSN, EBF1, PRKG1* (CAF); *CD1C, HLA-DPA1, HLA-DRB1, HLA-DRA, CD74, HLA-DPB1* (cDC2); *KRT14, DST, MMP7, MIR205HG, MT1X, OXTR, KRT17, FST* (basal/myoepithelial cells); *EPCAM, AMBP, MUC1* (epithelial cells); *TIMP1, FN1, POSTN, ACTA2, BST2, LY6D, COL6A1, SLC20A1, COL6A2, KRT16, CD9, S100A4, EMP1, LRRC8A, EPCAM, PDPN, ITGB1, PDGFRA, THY1* (fibroblasts); *BANK1, CD79A, CD74, MS4A1, MEF2C, CD19, CD79B* (B); *RNASE1, C1QA, C1QB, C1QC, SELENOP* (cDC1); *MUCL1, MGP, ERBB4, ANKRD30A, AZGP1,AGR2, STC2* (LM cells); *TPSB2, TPSAB1, CPA3, MS4A2, HPGDS,KIT, ENPP3* (mast cells); *PTGDS, FCHSD2, GPR183, NR3C1, TCF4* (DCs); *FCHSD2, GZMB, PTGDS, TCF4, GPR183, IL3RA, CLEC4C, NRP1, LILRA4, TLR7, TLR9, IRF7, GZMB* (pDC); *IGKC, IGLC3, IGLC2, IGHG1, IGHA1, IGHG3, IGHG4, IGHA2, FCRL5, TNFRSF17* (plasma); *TIMP1, COL1A1, COL1A2, COL3A1, SPARC, ANIN, CDCA3, TPX2, CDCA8, FAM64A, NUF2, BIRC5, CEP55, SKA1, KIF15, TTK, MELK, TOP2A, PBK, CCNA2, SPC25, MKI67* (cCAF); *PNPLA2, CAV1, FABP4, PPARG, CEBPA, LEP, CIDEA, SHOX2, SLC7A10, SLC36A2, P2RX5* (adipocytes); *ITGAM, LGALS3, CD68, CD163, LYZ, ADGRE1, LAMP2* (macrophages); *CD14, FCGR3A, FCGR1A* (monocytes); and *MYC, ESR1, AR, PGR, CDH1, AKT1, ERBB2, EPCAM, KRT8, KRT18, KRT19* (tumor, markers are also shared by benign breast duct cell types).

We further subdivided certain cell types into subtypes or cell states using the following: *IKZF2, TNFRSF18, FOXP3, CTLA4, IL7R, IL2RA* (T_reg_ cells); *GZMH, GZMB, GZMA, PRF1, IFNG, FASLG, LAMP1, CD8A, CD8B, CD3E, CD3D* (cytotoxic T cells); *GZMK* (pre-exhausted T cells); *VSIR, TIGIT, ICOS, EOMES, HAVCR2, PDCD1, BTLA, CD244, LAG3, CD160, CTLA4, CD96* (exhausted T cells); *CD69, CD28, CD44, DPP4* (activated T cells); high ribosomal gene expression (RPhigh CD4^+^ T cells); *IL1A, IL1B* (IL1A^+^ macrophages); high *TLR2* and *CD86 and low CD163 and MRC1* (M1 macrophages); high *CD163,* high *MRC1* (M2 macrophages); high *CD163, MRC1, TLR2* and *CD86* (M1/M2 macrophages); high *CD163* and low *MRC1* (M2 partial macrophages); *MKI67* (proliferation marker); *CD69, CCR7* (medium-low), *SELL* (medium-low) and *IL7R* (medium-low) (activated T cells). Note that T cell marker sets were clearer once subsetted and re-clustered.

##### snRNA-seq mouse cell type annotation

To annotate the mouse single-cell data the following markers were used: Epcam, Krt5, Acta2, Myh11, Krt14, Trp63, Krt17, Myl9 (basal); Areg, Cited1, Ly6d, Prlr, Esr1, Pgr, S100a6 (LMs); Kit, Aldh1a3, Cd14, Gabrp, Tspan8 (LP cells ); Birc5, Hmgb2, Stmn1, Mki67 (cycling cells); Ptprc, Fyb (immune); Col4a1, Sparc, Col4a2, Lamb1, Col5a2 (stroma); and Fabp4, Lpl, C4b, Mylk, Hk2, Slc4a4, Dio2, Vegfa (fibroblasts).

##### snRNA-seq PAM50 subtype assignment

For single-nuclei data, to overcome data sparseness, expression-based subtyping was performed at the cluster level. After cell type annotation of the combined Seurat object across all samples, a separate Seurat object was created comprising only tumor cells and was clustered again using the original Louvain algorithm and top 30 PCA dimensions via ‘FindNeighbors’ and ‘FindClusters’ (with parameters, resolution of 0.5) functions. Mean expression of the 50 genes used in the PAM50 algorithm were obtained per cluster. PAM50 subtype assignments were obtained for each of these cluster means in the same way as was conducted for bulk-RNA-seq data (without bootstrapping).

##### Gene regulatory networks

To infer gene regulatory networks of TFs, we used pySCENIC interface (v.0.11.2) from the SCENIC pipeline^107^. We applied SCENIC on SCT-normalized assay of sampled merged snRNA combo object, 500 cells sampled randomly per cell type of each sample. The first step of the SCENIC workflow is utilizing a regression per-target approach, GRNBoost2, to infer coexpression modules. We provided the list of unique TFs that are present in the JASPAR2020 db^108^ as input. Steps 2 and 3 of regulon prediction were run with default parameters and using RcisTarget hg38__refseq_r80 v.9 gene-motif ranking databases (10 kbp around the transcription start site (TSS) and 500 bp around TSS). Next, we recalculated the AUCell score, the regulon activity, to identify significant regulons (TFs) in each cell type. Then only regulons with at least 20 regulated genes were considered in the final heatmap. Finally, we generated a binary regulon activity heatmap to show gene regulatory networks relationships between TFs and their target genes using the ComplexHeatmap R package.

##### Mapping and quantification of snATAC-seq and snMultiome-seq

To process sequenced snATAC-seq and snMutiome-seq data, we used cellranger-atac count (v.2.0, 10x Genomics) and cellranger-arc count (v.2.0, 10x Genomics) pipelines, respectively. These pipelines filter and map snATAC-seq reads and identify transposase cut sites. The cellranger-arc count pipeline also performs filtering and alignment of snRNA-seq reads. The GRCh38 human reference was used for mapping reads.

##### Peak calling for snATAC-seq data

We performed peak calling using MACS2 (ref. ^109^). We removed peaks from the Y chromosome and peaks overlapping genomic regions containing ‘N’. All peaks were resized to 501 bp centered at the peak summit defined by MACS2. After this, we performed an iterative removal procedure described in Corces et al.^110^ to get the set of non-overlapping peaks. In brief, we started by retaining the most significant peak by MACS2 peak score (−log_10_(*q* value)) and removed all peaks that had a direct overlap with it. We repeated this procedure for the remaining peaks, until we had the set of non-overlapping peaks (sample peak set). The resulting set of sample peaks was used to calculate a peak-count matrix using FeatureMatrix from the Signac package v.1.3.0 (https://github.com/timoast/signac), which was also used for downstream analysis.

##### QC of snATAC-seq data

QC filtering of the snATAC-datasets was performed using functions from the Signac package. Filters that were applied for the cell calling include: number of fragments in peaks >1,000 and <20,000, percentage of reads in peaks >15, ENCODE blacklist regions percentage <0.05 (https://www.encodeproject.org/annotations/ENCSR636HFF/), nucleosome banding pattern score <10 and enrichment score for Tn5-integration events at transcriptional start sites >2. Peaks were annotated using R package ChiPseeker^111^ using the transcript database TxDb.Hsapiens.UCSC.hg38.knownGene. The promoter region was specified (−1,000,100) relative to the TSS.

##### Normalization, feature selection and dimension reduction of snATAC-seq data

The filtered peak-count matrix was normalized using term frequency-inverse document frequency (TF-IDF) normalization implemented in the Signac package. All peaks were used as features for dimensional reduction. We used the RunSVD Signac function to perform singular value decomposition on the normalized TF-IDF matrix, which is known as latent semantic indexing (LSI) dimension reduction. The resulting 2:30 LSI components were used for nonlinear dimension reduction using function RunUMAP from the Seurat package.

##### Clustering of snATAC-seq data

The nuclei were clustered using a graph-based clustering approach implemented in Seurat. First, we utilized the Seurat function FindNeighbors to construct a Shared Nearest Neighbor graph using the 2:30 LSI components. Next, we used the FindClusters function to iteratively group nuclei together while optimizing modularity using the Louvain algorithm.

##### Merging of snATAC-seq data across samples

Merging of snATAC-seq datasets was performed using functions from the Signac and Seurat packages. To normalize the peaks’ significance scores across samples, we converted MACS2 peak scores (−log_10_(*q* value)) to a score per million as described by Corces et al.^110^. To get the set of peaks for merging, we first combined peaks from all samples of the cohort. For overlapping peaks across samples, we performed an iterative removal procedure using normalized peak scores as described above. This yields the cohort level peak set. The resulting list of peaks was quantified and was used to create a peak-cell matrix so that the set of features was the same across all snATAC-seq samples. After that, the merge function from the Seurat package was used to merge snATAC-seq datasets. Next, we performed TF-IDF normalization. LSI-dimensional reduction was performed using the RunSVD function. Nonlinear dimension reduction was performed using the RunUMAP function with the first 2:50 LSI components.

##### Cell type label transfer from snRNA-seq to snATAC-seq data

Cell type label transfer was performed using functions from Signac and Seurat. First, we quantified chromatin accessibility associated with each gene by summing the reads overlapping the gene body and its upstream region of 2 kb, thus creating the gene by cell matrix. Coordinates for the genes were used from the Ensembl database v.86 (EnsDb.Hsapiens.v86 package). Next, we performed log-normalization of the resulting matrices using the NormaliseData function. The integration of paired snATAC-seq and snRNA-seq datasets was performed using the FindTransferAnchors function with the canonical correlation analysis option for the dimensional reduction. We then utilized the TransferData function to transfer cell type labels from the snRNA-seq dataset to the snATAC-seq dataset using the obtained set of anchors from the previous step. Then cell types were re-evaluated at the cohort-object level, where for each cluster the cell type label was assigned as the most abundant cell type in that cluster.

##### Identifying differentially accessible chromatin regions using snATAC-seq data

To identify differentially accessible chromatin regions, we used the FindMarkers function from the Seurat package with logistic regression test and the fraction of fragments peaks as a latent variable to reduce the effect of different sequencing depths across cells. Additionally, we also specified the following parameters: min.pct = 0.1, min.diff.pct = 0.1, logfc.threshold = 0 and only.pos = F. Bonferroni correction was applied for *P* value adjustment using all peaks from each comparison and peaks were considered significant if they had an adjusted *P* value <0.05.

##### Calculation of TF motif scores using snATAC-seq data

To evaluate TF binding accessibility profiles, we used the ChromVar tool^112^, which calculates bias-corrected deviations (TF motif scores) corresponding to gain or loss of accessibility for each TF motif relative to the average cell profile. To identify TFs with differential activity between cell groups of snATAC-seq data, we used a two-sided Wilcoxon rank-sum test for the whole set of TFs in the assay and applied FDR correction for the resulting *P* values. For subtype-specific TFs we additionally required them to have positive fold change (FC) in RNA-seq data when compared between tumor cells from each subtype versus pooled tumor cells from all other subtypes.

##### Identifying lineage-specific TF motifs in accessible chromatin regions using snATAC-seq data

To identify cancer lineage-specific motif profiles in open chromatin, we first compared tumor cells and their putative benign cell of origin versus all other epithelial cell groups (list of cell groups was basal-like BC, HER2-enriched BC, luminal A/B BC, LM cells, LP cells and basal myoepithelial cells) using a two-sided Wilcoxon rank-sum test followed by FDR correction, as above. Cancer-associated nonlineage-specific TFs were filtered out by excluding TFs enriched in both luminal BC versus LM cells and basal-like BC versus LP cells. Individual differential motif accessibility comparisons were conducted for each BC subtype and benign cell type versus all other epithelial cells and any TFs significantly enriched in a nonlineage cell type (with mean motif score difference of at least 0.5) were also excluded from the lineage-specific motif list.

##### Visualizing the coverage of snATAC-seq data

For snATAC-seq coverage plots, we used the CoveragePlot function from the Signac package. For tumor samples, we plotted coverage for tumor cells only and for normal cell populations we plotted coverage for combined cells across all samples.

##### Monocle pseudotime analysis using snATAC-seq data

Trajectory-based analyses of snATAC-seq data were performed using Monocle2 (ref. ^113^). To build case-level trajectories, we used subsetted data from the snATAC-seq merged object. For each case we subsetted 1,000 cells from the pool of its tumor cells and also 1,000 cells from each of the combined sets of normal cell populations, such as LM, basal/myoepithelial and LP cells. To create a Monocle cds object we used the function newCellDataSet on slot count and top 50,000 expressed peaks and with parameters lowerDetectionLimit = 0.5 and expressionFamily = negbinomial.size(). To estimate size factors for each cell and dispersion function for the peaks, we used functions estimateSizeFactors and estimateDispersions. Dimensionality reduction was performed using reduceDimension function with DDRTree method and max_component = 10.

##### scVarScan mutation mapping

We applied an in-house tool called scVarScan that can identify reads supporting the reference and variant alleles covering the variant site in each individual cell by tracing cell and molecular barcode information in an scRNA bam file. The tool is freely available at https://github.com/ding-lab/10Xmapping. For mapping, we used high-confidence somatic mutations from WES data.

##### CNV calling on bulk whole-exome data

Somatic copy number variants (CNVs) were called using GATK v.4.1.9.0114. The hg38 human reference genome (National Cancer Institute GDC data portal) was binned into target intervals using the PreprocessIntervals function, with a bin-length of 1,000 bp and the interval-merging-rule set to OVERLAPPING_ONLY. A PON was generated using each normal sample by utilizing the GATK functions CollectReadCounts with the argument–interval-merging-rule OVERLAPPING_ONLY, followed by CreateReadCountPanelOfNormals with the argument–minimum-interval-median-percentile 5.0.

For tumor samples, reads that overlapped the target interval were counted using the GATK function CollectReadCounts. Tumor read counts were standardized and de-noised using the GATK function DenoiseReadCounts, with the PON specified by–count-panel-of-normals. Allelic counts for tumor samples were generated for variants present in the af-only-gnomad.hg38.vcf file, following GATK best practices (variants further filtered to 0.2 > allele frequency > 0.01 and entries marked with ‘PASS’), using the GATK function CollectAllelicCounts. Segments were modeled using the GATK function ModelSegments, utilizing the de-noised copy ratio and tumor allelic counts as inputs. Copy ratios were called on the segment regions using the GATK function CallCopyRatioSegments.

To map the copy number ratios from segments to genes and assign amplifications or deletions, the Bedtools intersection^114^ was used. For genes overlapping multiple segments, a custom Python script was employed to call that gene as amplified, neutral or deleted based on a weighted copy number ratio calculated from copy ratios of each segment overlapped, the lengths of the overlaps and the *z*-score threshold used by the CallCopyRatioSegments function. If the resulting *z*-score cutoff fell within the range of the default *z*-score thresholds used by CallCopyRatioSegments (0.9, 1.1), the bounds of the default *z*-score threshold were utilized instead, replicating the logic of the CallCopyRatioSegments function. Similarly, to map copy number ratios from segments to chromosome arms, another script was used following the same approach to call whether the chromosome arm was amplified, neutral or deleted.

##### scRNA CNV detection

To detect large-scale chromosomal CNVs using scRNA-seq data, inferCNV (v.0.8.2) was used with default parameters recommended for 10x Genomics data. All cells that were not tumor were pooled together for the reference normal set. inferCNV was run at a sample level and only with post-QC filtered data.

##### Differential scRNA expression analyses

For cell-level and cluster-level differential expression, we used the ‘FindMarkers’ or ‘FindAllMarkers’ Seurat function as appropriate, with a minimum pct. of 0.25 and looking only in the positive direction, as lack of expression is harder to interpret due to the sparsity of the data. The resulting DEGs were then filtered for adjusted *P* < 0.05 and sorted by FC. All differential expression analyses were carried out using the ‘SCT’ assay.

##### Cell surface annotation

To annotate a given biomarker, we annotated each DEG by their subcellular location. Three databases were used to curate the subcellular location information: (1) Gene Ontology term 0005886; (2) Mass Spectrometric-Derived Cell Surface Protein Atlas^115^; and (3) The Human Protein Atlas subcellular location data based on The Human Protein Atlas v.19.3 and Ensembl v.92.38.

##### Identification of tumor markers using snRNA-seq data

To identify tumor cell surface markers, we used functions of the Seurat package. We compared gene expression between tumor cells and non-tumor cells across snRNA-seq samples of basal and luminal subtypes. The pipeline consists of the following steps: (1) compare expression levels across cell types per sample to identify tumor cell markers; and (2) select genes that were annotated as cell surface proteins by Gene Ontology (term 0005886). Using this approach, we identified candidate surface markers overexpressed in tumor cells compared to all the other cell types in a majority of individual samples (step 1) in each subtype. A gene was labeled tumor-cell-specific if both the following criteria were satisfied: (1) the average expression of the gene was higher in tumor cells compared to any other cell type for at least one sample and that all the differences were of statistical significance (log(FC) > 0; adjusted *P* value (*P*adj) < 0.05); (2) the average expression of the gene was higher in tumor cells compared to non-tumor cells (as a combined population) for 90% of the samples and that such differences were found to be statistically significant in at least 75% of the samples. All *P* values were adjusted by Bonferroni correction.

##### Receptor–ligand interactions

The CellPhoneDB Python package^52^ was used to find interactions between cell types in individual objects. Annotation and input counts files were constructed as previously described^52^. The statistical analysis method of the CellPhoneDB package was run with 1,000 iterations. Ligand–receptor pairs from the ‘significant means’ output file were used in the downstream analysis. Interactions were filtered by the number of cells belonging to an interacting cell type (>10) and by the percentage of interacting cells in the total number cells in a sample (>0.1%). Only interactions annotated as ‘curated’ were used for the analysis.

##### Differential gene expression analysis for subtype-specific transcriptional changes

We used the Seurat function FindMarkers (test.use = ‘LR’, only.pos = T, logfc.threshold = 0.2, min.pct = 0.1) to evaluate DEGs between epithelial cell types. First, we identified genes that were differentially expressed between related lineage groups against all other epithelial cell types. These included: (G1) basal-like tumor and LP; (G2) luminal A tumor, luminal B tumor, LM; and (G3) LP and LM. Second, intra-lineage DEGs were obtained for the following comparisons: (C1) basal-like tumor versus LP; and (C2) luminal A tumor and luminal B tumor versus LM. Finally, we extracted all DEGs (A1) for each epithelial cell type versus all other epithelial cells (FindAllMarkers). This analysis was performed only on snRNA-seq data. For analyzing the changes between basal tumors and luminal progenitors, we focused our analysis on genes identified in G1 with *P*adj < 0.05. We then filtered out genes that were found in C1 or C2 (cancer-related genes not related to lineage). We then filtered out genes in G2 (DEGs related to the luminal tumor transition). Using the all-DEG analysis (A1) we extracted DEGs specific to basal tumor or luminal progenitor and filtered out any genes specific to luminal A tumor, luminal B tumor, HER2 tumor, basal/myoepithelial and LM DEG analysis. Using this final gene list, we obtained the average expression across all epithelial cell types (focusing on genes with pct.exp > 20 and avg_log_2_FC > 1) for our final analysis. For analyzing the changes between luminal A/B tumors and LM cells we focused our analysis on genes identified in G1 with *P*adj < 0.05. We then filtered out genes that were found in C1 or C2, being only cancer type related changes and not related to cell of origin. We then filtered out genes in G1, DEGs related to the basal-like tumor transition. Using the all-DEG analysis (A1) we extracted DEGs specific to luminal A tumor, luminal B tumor or LM and filtered out any genes specific to HER2 tumor, basal/myoepithelial or basal-like tumor DEG analysis. Using this final gene list, we extracted the average expression across all epithelial cell types (focusing on genes with pct.exp > 20 and avg_log_2_FC > 1) for our final analysis.

##### Analysis of lymphocyte-dense clusters in ST data

For each ST spot overlapping a lymphocyte-dense region (Extended Data Fig. 2), spots were subset from the RDS object for each sample and merged using the Seurat merge function. At least three lymphocyte-dense regions were identified on each slide.

##### Spatial mapping of snRNA-seq cell types to ST data

We generated a joint cell type reference Seurat object for each PAM50 subtype by merging snRNA-seq data from the same subtype (basal, luminal A, luminal B and Her2). Using these snRNA-seq references, we then inferred the cell type composition in ST samples of the same PAM50 subtype using CytoSPACE^53^, a tool that aligns snRNA-seq to ST data and resolved cell type compositions per spatial spot. A custom script was developed to facilitate preprocessing the snRNA-seq and ST file, as well as integrating the CytoSPACE result into the Seurat object for easier downstream analysis and visualization. These workflow and processing scripts can be found in the GitHub page associated with this manuscript (https://github.com/ding-lab/HTAN_BRCA_publication).

#### CODEX quantification and analysis

##### Multiplex image segmentation

Multiplex images were segmented using the Mesmer pretrained nuclei + membrane segmentation model in the Deepcell^116^ cell segmentation library. The 4,6-diamidino-2-phenylindole (DAPI) channel was used as the nuclei segmentation image and pan-cytokeratin, E-cadherin, CD45, CD8, CD3, Vimentin, SMA, CD31 and CD20 channels were merged and used as the membrane segmentation image. Following segmentation, cells were classified as positive or negative for the following epithelial markers: GATA3, c-Kit, CK14, CK19, ER, PR and Her2. To eliminate batch effects, marker thresholds were set manually for each image.

##### Normal/tumor region identification and classification

We then identified epithelial regions in an unsupervised fashion. First, pan-cytokeratin and E-cadherin were thresholded on the values discussed in the section above. The masks were then merged into a consensus mask. This mask was passed through a Gaussian filter (sigma = 2.0) and hole-filling algorithm. The regions in the resulting mask (*n* = 12,513 across all images) were then classified into normal and tumor regions.

A pseudo-color RGB image was generated for each region that represented image intensities for pan-cytokeratin, SMA, DAPI and podoplanin. A subset of these regions (*n* = 637) was then manually annotated as normal, ductal carcinoma in situ, invasive ductal carcinoma or image artifacts. These annotations were then partitioned via an 80:20 split into training and validation datasets. During model training, images were augmented by random affine rotation and color jitter. A convolutional neural network was used to classify these images based on region type. The neural network consisted of two ResNet34 stems (one for the three-channel pseudo-color RGB image and another for a one-channel mask representing the pixels the bounds of the region in the pseudo-color RGB image), the stems were then merged into a prediction head consisting of three linear layers separated by ReLU activation functions and batch normalization layers. The final layer was followed by a Softmax activation that outputs classification probabilities. The network was trained for 500 epochs and achieved a validation accuracy of 96%. The network was then used to predict all 12,513 regions. All regions predicted by the model were then manually reviewed to further reduce the region annotation error rate. In total, 649 regions were annotated as normal and 10,753 as tumor. Images classified as image artifacts were excluded from downstream analysis.

##### Epithelial marker comparison among normal and tumor regions across subtypes

For epithelial marker comparisons, the fraction of positive cells within each tumor region for each epithelial marker was calculated. Values for each region were then averaged at the sample level before plotting and significance calculations.

#### Immunofluorescence quantification and analysis

Immunofluorescence images were first standardized by considering only the first 1,250 lines of each image. To quantify the MELK signal, a mask based on E-cadherin was generated by adaptive thresholding. For each sample, the threshold value was set to half of the E-cadherin sample mean. The mask was then generated as follows: for a given sample, it was set to one where the E-cadherin value was above the threshold and set to zero elsewhere. The average MELK pixel intensity per sample was then calculated considering only the positive regions of the mask. Finally, the resulting averages were grouped by their case (five each) and the results were displayed in a violin plot. Corresponding *P* values between all basal-like and luminal cases were calculated.

### Reporting summary

Further information on research design is available in the Nature Portfolio Reporting Summary linked to this article.

## Supplementary information

Reporting Summary

Supplementary Table 1 Supplementary Tables 1–6.

## Source data

Source Data Fig. 3 Cell type composition for snRNA, scRNA and ST data related to CD8 exhausted cells.

Source Data Fig. 5 Positive cell fractions associated with codex antibody staining across all markers in Fig. 5.

Source Data Extended Data Fig. 1 Average nFeature RNA and number of cells by sample for each study in the comparison.

Source Data Extended Data Fig. 2 Proportion of cell types by sample for indicated T cells.

Source Data Extended Data Fig. 4 Cell type compositions associated with ST data.

Source Data Extended Data Fig. 10 Intensity values for all samples indicated in Extended Data Fig. 10.

## Data Availability

All human sequencing and imaging data have been deposited via the WU-HTAN dbGaP study accession phs002371.v1.p1 (https://www.ncbi.nlm.nih.gov/projects/gap/cgi-bin/study.cgi?study_id=phs002371.v1.p1). In addition, all data have been deposited to the HTAN Data Coordinating Center Data Portal at the National Cancer Institute at https://data.humantumoratlas.org/ (under the HTAN WUSTL Atlas). All mouse snRNA/snATAC data have been deposited to the Gene Expression Omnibus under series GSE240577. Source data are provided with this paper.

## Code availability

Scripts related to specific analysis in the paper can be found on GitHub at https://github.com/ding-lab/HTAN_BRCA_publication/tree/main.
