# Supplementary material for: Differential chromatin accessibility and transcriptional dynamics define breast cancer subtypes and their lineages
Source: Nat Cancer. 2024 Oct 30;5(11):1713–36. doi: 10.1038/s43018-024-00773-6 (PMC11584403; doi:10.1038/s43018-024-00773-6)
Supplement: Supplementary file 1 — Reporting Summary [file 43018_2024_773_MOESM1_ESM.pdf]

Corresponding author(s): William E. Gillanders and Li Ding

Last updated by author(s): 01/10/2024

Reporting Summary

Nature Portfolio wishes to improve the reproducibility of the work that we publish. This form provides structure for consistency and transparency in reporting. For further information on Nature Portfolio policies, see our Editorial Policies and the Editorial Policy Checklist.

Statistics

For all statistical analyses, confirm that the following items are present in the figure legend, table legend, main text, or Methods section.

n/a	Confirmed	
☐	☒	The exact sample size (*n*) for each experimental group/condition, given as a discrete number and unit of measurement
☐	☒	A statement on whether measurements were taken from distinct samples or whether the same sample was measured repeatedly
☐	☒	The statistical test(s) used AND whether they are one- or two-sided<br>*Only common tests should be described solely by name; describe more complex techniques in the Methods section.*
☐	☒	A description of all covariates tested
☐	☒	A description of any assumptions or corrections, such as tests of normality and adjustment for multiple comparisons
☐	☒	A full description of the statistical parameters including central tendency (e.g. means) or other basic estimates (e.g. regression coefficient) AND variation (e.g. standard deviation) or associated estimates of uncertainty (e.g. confidence intervals)
☐	☒	For null hypothesis testing, the test statistic (e.g. *F*, *t*, *r*) with confidence intervals, effect sizes, degrees of freedom and *P* value noted<br>*Give P values as exact values whenever suitable.*
☒	☐	For Bayesian analysis, information on the choice of priors and Markov chain Monte Carlo settings
☒	☐	For hierarchical and complex designs, identification of the appropriate level for tests and full reporting of outcomes
☐	☒	Estimates of effect sizes (e.g. Cohen's *d*, Pearson's *r*), indicating how they were calculated

Our web collection on statistics for biologists contains articles on many of the points above.

Software and code

Policy information about availability of computer code

Data collection

No software was used for data collection.

Data analysis

Scripts related to specific analysis in the paper can be found at the following github link: https://github.com/ding-lab/HTAN_BRCA_publication/tree/main
Ascore v1.0.6858, (Beausoleil et al., 2006), https://github.com/PNNL-Comp-Mass-Spec/AScore
bam-readcount v0.8, McDonnell Genome Institute, https://github.com/genome/bam-readcount
Bioconductor v3.9, (Huber et al., 2015), https://bioconductor.org/
CellPhoneDB, (Efremova et al., 2020), https://www.cellphonedb.org
Cell Ranger v3.1.0, 10X Genomics, https://support.10xgenomics.com/single-cell-gene-expression/software/pipelines/latest/what-is-cell-ranger
Space Ranger v1.1.0, 10X Genomics, https://support.10xgenomics.com/spatial-gene-expression/software/pipelines/latest/what-is-space-ranger
gnomAD (r2.1.1), https://gnomad.broadinstitute.org/
CharGer v0.5.4, (Scott et al., 2019), https://github.com/ding-lab/CharGer
clusterProfiler v3.8.1, (Yu et al., 2012), https://bioconductor.org/packages/release/bioc/html/clusterProfiler.html
ConsensusClusterPlus v1.56.0, (Wilkerson and Hayes, 2010), https://bioconductor.org/packages/release/bioc/html/ConsensusClusterPlus.html
data.table_1.12.6 , R Development Core Team, https://cran.r-project.org/package=data.table
dendsort_0.3.3, (Sakai et al., 2014), https://cran.r-project.org/package=dendsort
dplyr_0.8.5, R Development Core Team, https://cran.r-project.org/package=dplyr
ESTIMATE, (Yoshihara et al., 2013), https://sourceforge.net/projects/estimateproject/
GATK v4.0.0.0, (McKenna et al., 2010), https://github.com/broadgsa/gatk
germlinewrapper v1.1, Li Ding Lab, https://github.com/ding-lab/germlinewrapper

ggplot2_3.3.2, R Development Core Team, https://CRAN.R-project.org/package=ggplot2
gridExtra_2.3, R Development Core Team, https://cran.r-project.org/package=gridExtra
bulk-RNA-Seq expression, Li Ding Lab, https://github.com/ding-lab/HTAN_bulkRNA_expression
inferCNV v0.8.2, (Tickle et al., 2019), https://github.com/broadinstitute/infercnv
Integrative Genomics Viewer, (Robinson et al., 2011), https://igv.org
Loupe Browser v.5.0, 10X genomics, https://www.10xgenomics.com/products/loupe-browser
magrittr_1.5, R Development Core Team, https://cran.r-project.org/package=magrittr
Matrix_1.2-17 , R Development Core Team, https://CRAN.R-project.org/package=Matrix
MEDALT, (Wang et al., 2020), https://github.com/KChen-lab/MEDALT
MuTect v1.1.7, (Cibulskis et al., 2013), https://github.com/broadinstitute/mutect
pheatmap_1.0.12, R Development Core Team, https://cran.r-project.org/package=pheatmap
Pindel v0.2.5, (Ye et al., 2009), https://github.com/genome/pindel
Python v3.7, Python Software Foundation, https://www.python.org/
R v3.6, R Development Core Team, https://www.r-project.org/
RColorBrewer_1.1-2, R Development Core Team, https://CRAN.R-project.org/package=RColorBrewer
RCTD v.1.2.0, (Cable et al., 2021), https://github.com/dmcable/RCTD
reshape2_1.4.3, R Development Core Team, https://cran.r-project.org/package=reshape2
Samtools v1.2, (Li et al., 2009), https://www.htslib.org/
scVarScan, Li Ding Lab, https://github.com/ding-lab/10Xmapping
SeqQEst, Li Ding Lab, https://github.com/ding-lab/SeqQEst
Seurat v3.1.2 and v4.0.3, (Butler et al., 2018), https://cran.r-project.org/web/packages/Seurat
somaticwrapper v1.5, Li Ding Lab, https://github.com/ding-lab/somaticwrapper
sva v3.40.0, (Huber et al., 2015), https://bioconductor.org/packages/release/bioc/html/sva.html
STAR v2.7.4a, (Dobin et al., 2013), https://github.com/alexdobin/STAR
Strelka v2.9.2, (Kim et al., 2018), https://github.com/Illumina/strelka
stringr_1.4.0, R Development Core Team, https://cran.r-project.org/package=stringr
Subread v2.0.1, (Liao et al., 2013), https://sourceforge.net/projects/subread/
Tidyverse, (Wickham et al., 2019), https://www.tidyverse.org/
VarScan v2.3.8, (Koboldt et al., 2012), https://dkoboldt.github.io/varscan/
viridis_0.5.1, R Development Core Team, https://github.com/sjmgarnier/viridis
viridisLite_0.3.0, R Development Core Team, https://github.com/sjmgarnier/viridis
xCell v1.2, (Aran et al., 2017), http://xCell.ucsf.edu/
Monocle3 v3.10, https://cole-trapnell-lab.github.io/monocle3/docs/installation/
CopyKat 1.0.4 (Gao et al., 2021) ,https://github.com/navinlabcode/copykat
inferCNV post processing, https://github.com/ding-lab/infer_cnv_postprocesssing.git
AUCell v1.19.1, R-package
bulk-RNA-seq expression, https://github.com/ding-lab/HTAN_bulkRNA_expression
Cell Ranger v6.0.2, 10X Genomics
Cell Ranger ATAC v2.0, 10X Genomics
Cell Ranger ARC v2.0, 10X Genomics
ChIPseeker v1.26.2, R-package
chromVAR v1.12.0, R-package
COCOON, https://github.com/ding-lab/COCOONS
ComplexHeatmap v2.6.2, R-package
GATK v4.0.0.0, https://github.com/broadgsa/gatk
ggdendro v0.1.22, R-package
igraph v1.2.6, R-package
InferCNV v0.99.7 and v1.11.2, https://github.com/broadinstitute/infercnv
MACS2 v2.2.7.1, https://github.com/macs3-project/MACS
MuTect v1.1.7 and MuTect2 v4.1.2.0, https://github.com/broadinstitute/mutect
motifmatchr v1.12.0, R-package
Pindel v0.2.5, https://github.com/genome/pindel
pySCENIC v0.11.2, https://pyscenic.readthedocs.io/en/latest/index.html
Scrublet v0.2.3, https://github.com/swolock/scrublet
scVarScan, https://github.com/ding-lab/10Xmapping
Seurat v4.0.5, R-package
Signac v1.3.0 and v.1.4.0, R-package
sklearn v0.24.2, python-package
slingshot, v2.5.1, R-package
Somaticwrapper v1.6, https://github.com/ding-lab/somaticwrapper
Space Ranger v1.3.0, 10X Genomics
survival v3.2.7, R-package
Strelka v2.9.2, https://github.com/Illumina/strelka
VarScan v2.3.8, https://dkoboldt.github.io/varscan
ctc v1.54.0, R-package
heatmap.plus v.1.3, R-package
deepcell v0.12.6, https://github.com/vanvalenlab/deepcell-tf

For manuscripts utilizing custom algorithms or software that are central to the research but not yet described in published literature, software must be made available to editors and reviewers. We strongly encourage code deposition in a community repository (e.g. GitHub). See the Nature Portfolio guidelines for submitting code & software for further information.

Data

Policy information about availability of data

All manuscripts must include a data availability statement. This statement should provide the following information, where applicable:

- Accession codes, unique identifiers, or web links for publicly available datasets
- A description of any restrictions on data availability
- For clinical datasets or third party data, please ensure that the statement adheres to our policy

All human sequencing and imaging data have been deposited via the Human Tumor Atlas Network (HTAN) dbGaP Study Accession: phs002371.v1.p1 (https://www.ncbi.nlm.nih.gov/projects/gap/cgi-bin/study.cgi?study_id=phs002371.v1.p1). In addition, all data have been deposited to the HTAN Data Coordinating Center Data Portal at the National Cancer Institute: https://data.humantumoratlas.org/ (under the HTAN WUSTL Atlas). The human genome reference (hg38) can be found at the 10x site (https://www.10xgenomics.com/support/software/cell-ranger/downloads).

Human research participants

Policy information about studies involving human research participants and Sex and Gender in Research.

Reporting on sex and gender	Sex and gender were not considered in the study design.
Population characteristics	All patients involved in this study were between the ages of 30 and 89 with breast carcinoma. Patients were either treatment-naïve or had neoadjuvant therapy prior to sample collection. Detailed clinical information can be found in Supplementary Table 1.
Recruitment	Participants for this study were recruited from all patients undergoing surgical resection of breast cancer, and were screened only on the basis of resectable disease identified by the schedule contained within the electronic health record system (EPIC). Clinical research staff conducted a manual review to ensure each participant was assigned a number and consented at the Washington University in St. Louis, Department of Surgery and Barnes Jewish Hospital or on the day of surgery. All patients provided informed consent approved by the Washington University in St. Louis Institutional Review Board.
Ethics oversight	Tissue samples were collected with written consent from all patients under the protocol 11-08117 with approval from the Washington University in St. Louis Institutional Review Board. Consent included the use of all de-identified patient data for publication. Participants were not compensated.

Note that full information on the approval of the study protocol must also be provided in the manuscript.

Field-specific reporting

Please select the one below that is the best fit for your research. If you are not sure, read the appropriate sections before making your selection.

☒ Life sciences ☐ Behavioural & social sciences ☐ Ecological, evolutionary & environmental sciences

For a reference copy of the document with all sections, see nature.com/documents/nr-reporting-summary-flat.pdf

Life sciences study design

All studies must disclose on these points even when the disclosure is negative.

Sample size	Tumor samples were collected as patients underwent surgery during the collection time period on a rolling basis. The sample size in the manuscript is comparable or greater than most studies published to date. Tumor samples were collected as patients underwent surgery during the collection time period on a rolling basis, and thus no sample size was calculated. As the goal was to build an atlas, rather than to obtain sufficient statistical power, we included as many samples as we could up until manuscript submission.
Data exclusions	No data were excluded from the analysis.
Replication	We validated results using orthogonal technologies such as immunofluoresence staining, Codex, and spatial transcriptomics. Generally samples were not replicated but multiple fields of view were analyzed for consistency. General data quality was evaluated for all data presented in this study. Many samples were evaluated using multiple technologies to confirm key findings.
Randomization	Due to our patient accrual on a rolling basis, we did not 'allocate' patients into specific treatment groups, thus the imbalance in the number of patients from each subtype. Moreover, the study was designed around discovery and characterization, rather than clinical comparison of clinical subtypes or treatment arms. Consequently, there was no issue of controlling for covariates to reduce statistical noise for improving regression.
Blinding	Blinding was not relevant in our study as samples were collected from patients as they came into the clinic. As every sample was included in the study and processed in the same standardized way, knowledge of clinical phenotypes would not affect the study.

Reporting for specific materials, systems and methods

We require information from authors about some types of materials, experimental systems and methods used in many studies. Here, indicate whether each material, system or method listed is relevant to your study. If you are not sure if a list item applies to your research, read the appropriate section before selecting a response.

Materials & experimental systems

n/a	Involved in the study
☐	☒ Antibodies
☒	☐ Eukaryotic cell lines
☒	☐ Palaeontology and archaeology
☒	☐ Animals and other organisms
☒	☐ Clinical data
☒	☐ Dual use research of concern

Methods

n/a	Involved in the study
☒	☐ ChIP-seq
☒	☐ Flow cytometry
☒	☐ MRI-based neuroimaging

Antibodies

Antibodies used

Marker	Cell type(s)	Manufacturer	Catalog	Dilution
GATA3	Luminal mature	Akoya	4250085	1:100
ckit	Luminal progenitor	Abcam	ab216450	1:50
CK14	Basal/myoepithelial	Akoya	4450031	1:800
CK17	Tumor cells	Abcam	ab239986	1:50
CK19	Tumor cells	Abcam	ab195872	1:200
ER	Hormone receptor	Akoya	4250074	1:100
PR	Hormone receptor	Abcam	ab239793	1:100
GLUT1	Tumor cells	Abcam	ab196357	1:100
Histone H3 Phospho	Tumor cells	Akoya	4550115	1:800
CD31	Endothelium	Akoya	4450017	1:100
CD36	Adipocytes	Cell signaling	39914	1:50
E-Cadherin	Epithelium	Akoya	4250021	1:400
PanCytokeratin	Epithelium	Akoya	4450020	1:200
Podoplanin	Lymphatic endothelium	Akoya	4250004	1:400
SMA	Fibroblasts	eBiosciences	MA1-06110	1:100
Vimentin	mesenchymal marker	Akoya	4450050	1:100
Ki67	Proliferating cells	Akoya	4250019	1:800
CD3e	T cells	Akoya	4550119	1:100
CD4	CD4+T	Akoya	4550112	1:100
CD8	CD8+T	Akoya	4250012	1:800
CD68	Macrophage	Akoya	4550113	1:200
CD20	B cells	Abcam	ab236434	1:100
HLA-DR	APCs	Akoya	4550118	1:100
ERBB2	Tumor cells	Abcam	ab194979	1:150
MELK	Tumor cells	Thermofisher	MA517120	1:200
Maspin	Basal progenitor cell	Invitrogen	PA5-35104	1:100

Validation

Marker	Cell type(s)	Manufacturer	Catalog	Supporting references (PMID or URL)
GATA3	Luminal mature	Akoya	4250085	https://www.akoyabio.com/phenocycler/assays/
ckit	Luminal progenitor	Abcam	ab216450	32474164
CK14	Basal/myoepithelial	Akoya	4450031	https://www.akoyabio.com/phenocycler/assays/
CK17	Tumor cells	Abcam	ab239986	33462395
CK19	Tumor cells	Abcam	ab195872	33950524
ER	Hormone receptor	Akoya	4250074	https://www.akoyabio.com/phenocycler/assays/
PR	Hormone receptor	Abcam	ab239793	33579355
GLUT1	Tumor cells	Abcam	ab196357	32399910
Histone H3 Phospho	Tumor cells	Akoya	4550115	https://www.akoyabio.com/phenocycler/assays/
CD31	Endothelium	Akoya	4450017	https://www.akoyabio.com/phenocycler/assays/
CD36	Adipocytes	Cell signaling	39914	35854007
E-Cadherin	Epithelium	Akoya	4250021	https://www.akoyabio.com/phenocycler/assays/
PanCytokeratin	Epithelium	Akoya	4450020	https://www.akoyabio.com/phenocycler/assays/
Podoplanin	Lymphatic endothelium	Akoya	4250004	https://www.akoyabio.com/phenocycler/assays/
SMA	Fibroblasts	eBiosciences	MA1-06110	24797069
Vimentin	mesenchymal marker	Akoya	4450050	https://www.akoyabio.com/phenocycler/assays/
Ki67	Proliferating cells	Akoya	4250019	https://www.akoyabio.com/phenocycler/assays/
CD3e	T cells	Akoya	4550119	https://www.akoyabio.com/phenocycler/assays/
CD4	CD4+T	Akoya	4550112	https://www.akoyabio.com/phenocycler/assays/
CD8	CD8+T	Akoya	4250012	https://www.akoyabio.com/phenocycler/assays/
CD68	Macrophage	Akoya	4550113	https://www.akoyabio.com/phenocycler/assays/
CD20	B cells	Abcam	ab236434	32488028

HLA-DR	APCs	Akoya	4550118	https://www.akoyabio.com/phenocycler/assays/
ERBB2	Tumor cells	Abcam	ab194979	34824220
MELK	Tumor cells	Thermofisher	MA517120	https://www.thermofisher.com/antibody/product/MELK-Antibody-clone-2G2-Monoclonal/MA5-17120
